# Characterization of Hepatitis B Virus Transcripts in Chronically HBV-Infected Chimpanzees and Patients Treated with ARC-520 siRNA Demonstrates Transcriptional Silencing of cccDNA

**DOI:** 10.3390/v16121943

**Published:** 2024-12-19

**Authors:** Christine I. Wooddell, Dean Sanders, Zhao Xu, Lung-Yi Mak, Thomas Schluep, Wai-Kay Seto, Bruce D. Given, Man-Fung Yuen

**Affiliations:** 1 Arrowhead Pharmaceuticals Inc., 502 S. Rosa Road, Madison, WI 53719, USA; dsanders@arrowheadpharma.com; 2 Arrowhead Pharmaceuticals Inc., 10102 Hoyt Park Drive, San Diego, CA 92131, USA; zxu@arrowheadpharma.com; 3 Department of Medicine & State Key Laboratory of Liver Research, School of Clinical Medicine, The University of Hong Kong, Hong Kong, China; loeymak@gmail.com (L.-Y.M.); wkseto@hku.hk (W.-K.S.); mfyuen@hku.hk (M.-F.Y.); 4 Arrowhead Pharmaceuticals Inc., 177 E. Colorado Boulevard, Suite 700, Pasadena, CA 91105, USA; tschluep@arrowheadpharma.com (T.S.); bgiven@arrowheadpharma.com (B.D.G.)

**Keywords:** hepatitis B virus transcription, hepatitis B virus RNA, HBV X, cccDNA, chronic hepatitis B, HBV siRNA, RNA interference therapeutics

## Abstract

Full-length hepatitis B virus (HBV) transcripts of chimpanzees and patients treated with multidose (MD) HBV siRNA ARC-520 and entecavir (ETV) were characterized by single-molecule real-time (SMRT) sequencing, identifying multiple types of transcripts with the potential to encode HBx, HBsAg, HBeAg, core, and polymerase, as well as transcripts likely to be derived from dimers of dslDNA, and these differed between HBeAg-positive (HBeAg+) and HBeAg-negative (HBeAg−) individuals. HBV transcripts from the last follow-up ~30 months post-ARC-520 treatment were categorized from one HBeAg+ (one of two previously highly viremic patients that became HBeAg− upon treatment and had greatly reduced cccDNA products) and four HBeAg− patients. The previously HBeAg+ patient received a biopsy that revealed that he had 3.4 copies/cell cccDNA (two to three orders of magnitude more cccDNA than HBeAg− chimpanzees) but expressed primarily truncated X and HBsAg from iDNA, like two patients that were HBeAg− at the start of the study and had one copy/cell cccDNA. No HBV transcripts were detected in two other HBeAg− patients that had ~0.3 copies/cell cccDNA, one of which had seroconverted for HBsAg. The paucity of cccDNA-derived transcripts in the presence of high cccDNA demonstrates the transcriptional silencing of HBV following MD siRNA treatment with ETV.

## 1. Introduction

**Figure 1:**
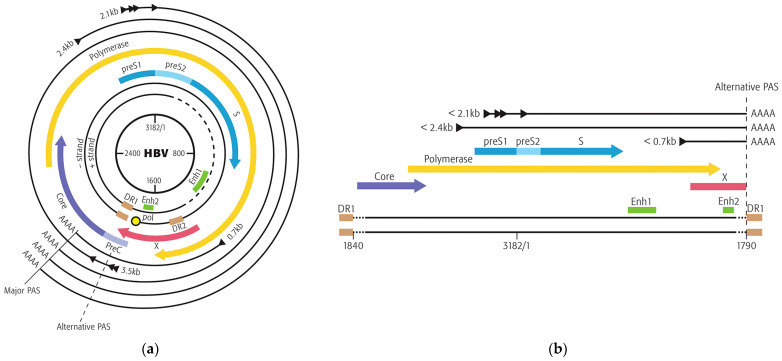
Schematic of the hepatitis B virus genome and transcripts from cccDNA or integrated double-stranded linear DNA. (**a**) The major HBV transcripts of cccDNA are ~3.5 kb for precore (HBeAg) and pgRNA (encodes core and HBV polymerase); 2.4 kb for pre-S1 (encodes L-HBsAg); 2.1 kb for pre-S2 (encodes M-HBsAg) and S (encodes S-HBsAg); and 0.7 kb for HBx. Open reading frames (ORF) are indicated for precore/core, polymerase, HBV S antigens (preS1 for L-HBsAg, preS2 for M-HBsAg, and S for S-HBsAg), and HBx. Positions of enhancers Enh1 and Enh2 (green) and Direct Repeats DR1 and DR2 (brown) are indicated. Transcripts of cccDNA utilize the major polyadenylation signal (PAS). (**b**) The major HBV transcripts of integrated HBV dslDNA (iDNA) are HBsAg and truncated HBx, though the ORF of core and polymerase may be intact. Transcripts of iDNA utilize either the alternative HBV PAS or a PAS in the integrated host DNA. Reproduced from [4] with permission from Elsevier.

Despite the availability of an effective hepatitis B virus (HBV) vaccine, there are an estimated 316 million people chronically infected with HBV, accounting for more than 0.5 million deaths due to HBV-related complications [1]. Infection is maintained by the double-stranded genome of HBV that resides in the nucleus of infected hepatocytes as a 3.2-kilobase (kb) covalently closed circular DNA (cccDNA) minichromosome. Four promoter regions produce transcripts that are approximately 3.5 kb, 2.4 kb, 2.1 kb, and 0.7 kb [2]. As shown in Figure 1a, the HBV genome encodes the precore protein (HBeAg), the hepatitis B surface antigen (HBsAg), comprised of large (L), middle (M), and small (S) S proteins that form the envelope of the HBV virion, the HBV X protein (HBx), and the pregenomic RNA (pgRNA), which itself encodes core and HBV polymerase (pol). Cellular RNA polymerase II transcribes all the cccDNA-derived transcripts (messenger RNA, mRNA) encoding these HBV proteins, which are encoded in overlapping reading frames and terminate at the same nonconventional major HBV polyadenylation signal (PAS) 5′-UAUAAA-3′ [3].

Precore mRNA and pgRNA are transcribed from the same basal core promoter but utilize different translation start codons for HBeAg and core, resulting in different but overlapping and in-frame open reading frames (ORF) [5]. The 2.4 kb pre-S1 transcript encodes L-HBsAg, and the 2.1 kb pre-S2/S transcripts encode the less abundant M-HBsAg and highly abundant S-HBsAg utilizing two separate start codons [3,6].

Despite the difficulties of studying HBx, as reviewed by Slagle and Bouchard, an abundance of HBx studies demonstrated its ability to modulate transcription and disrupt cellular processes [7]. HBx is a regulatory protein essential to initiate and maintain the transcription of cccDNA through blocking the structural maintenance of chromosome 5/6 complex (SMC5/6) [8,9,10,11,12]. Importantly, Allweiss et al. demonstrated in HBV-infected human hepatocyte chimeric mice that siRNA-induced reduction in HBV proteins including HBx restored SMC5/6 [13]. Using a full-length 5′RACE method, Stadelmayer et al. detected in HepG2-NTCP cells the 0.7 kb canonical X transcripts that initiated between HBV positions 1243–1338, relative to the start codon at 1374, and would be expected to produce the 154 amino acid HBx protein, and also shorter X transcripts that initiated after the first ATG and could presumably be translated from one of the two ATG codons further downstream (Met79/Met103) [14]. Both the canonical X transcripts and the short X transcripts were observed in primary human hepatocytes, and in addition there were longer X transcripts that initiated from positions 1065–1151. A major transcription start site at 1523 and minor transcription start sites within the X open reading frame (ORF) were previously characterized using cap analysis of gene expression of HCC/nontumor liver sample pairs from sixteen CHB patients [15].

HBV DNA integration is a by-product of the replication process resulting from a shorter double-stranded linear DNA (dslDNA, Figure 1b) product with Direct Repeat 1 (DR1) at both ends that forms instead of rcDNA with its 7–9 base terminal redundancy [16]. Linear dslDNA is prone to integrate and tends to lose some sequences from both ends in so doing. The truncated ends of the dslDNA exclude the precore/core promoter and, thus, iDNA does not produce HBeAg nor pgRNA but can produce HBsAg and truncated X transcripts [17,18,19,20,21]. Hence, chronically HBV-infected individuals produce HBsAg transcripts from both the cccDNA and from HBV DNA sequences that integrate into the host genome (iDNA).

HBsAg produced from iDNA led to differential HBsAg responses between chronically HBV-infected HBeAg-positive (HBeAg+) and HBeAg-negative (HBeAg−) patients and chimpanzees treated with the HBV siRNA therapeutic ARC-520 previously in development along with the nucleoside analog (NA) entecavir (ETV) [17,22,23]. ARC-520 was comprised of two siRNAs named “siHBV-74” and “siHBV-77” [24]. The binding site for siHBV-74 was position 1779–1797 and the binding site for siHBV-77 was 1825–1843, relative to GenBank sequence V01460 that has DR1 at 1826–1836. Thus, both siRNAs target sequences near the HBV DR1 element, a highly conserved region of HBV, but a region often deleted from integrated dslDNA. An in silico sequence conservation analysis of these siRNA sequences predicted that together, these two siRNAs would perfectly match 99.6% of all HBV genomes [24]. However, HBsAg transcripts expressed from iDNA commonly lack these siRNA binding sites and, thus, resulted in the limited reduction in HBsAg in response to ARC-520. This point was apparent in the HBeAg+ patients of the clinical study Heparc-2001 who had been on ETV prior to the study. In these patients, HBeAg and HBV core-related antigen (HBcrAg) were reduced by 1.2 log_10_ and 0.9 log_10_ at nadir, respectively, demonstrating that the precore and core transcripts (transcribed from cccDNA) responded significantly more to the ARC-520 siRNAs than the S transcripts that were reduced by only 0.3 log_10_ IU/mL. In HBeAg+ patients who were previously NA-naïve, however, HBsAg was reduced by a mean of 1.4 log_10_ IU/mL from a single dose of ARC-520, indicating that much more of the HBsAg in these patients was derived from cccDNA. The molecular characterization of these HBeAg+ and HBeAg− features was performed with samples from chronically HBV-infected chimpanzees.

Other groups corroborated our conclusions regarding integrated HBV DNA producing HBsAg. Using an RT-PCR approach with liver tissues from five CHB patients, Freitas et al. demonstrated the relative abundance of integrant-derived transcripts that could produce HBsAg [18]. Meier et al. concluded from the molecular analysis of liver biopsies from HBeAg− patients with low viral loads that transcriptionally active HBV integration can extend to the entire liver in some HBeAg− patients [19]. HBsAg detected via immunohistochemistry and HBV transcripts detected via in situ hybridization revealed that nearly all the hepatocytes in these patients produced HBsAg, despite the low viral load. The molecular analysis of the intra-hepatic viral RNA confirmed the virus–host junctions in all the HBeAg− CHB patients.

Heparc-2001 was a phase 2 clinical study to determine the safety and tolerability of ARC-520 and to assess its effects on the virological parameters of HBeAg+ and HBeAg− CHB patients [17]. A cohort of eight patients from Heparc-2001, who prior to the study had been treatment-naïve, received multidose (MD) HBV siRNA ARC-520 (four to nine doses) with ETV and had long-term follow-up of approximately 30 months [23]. The serological, virological, and histological responses to RNA inhibition by ARC-520 were previously characterized [23]. Five patients who consented to liver biopsy at the last follow-up at an average of 30 weeks after the final ARC-520 dose had sufficient liver RNA to characterize the post-ARC-520 HBV transcripts by single-molecule real-time (SMRT) sequencing (Iso-seq), the results of which are reported here for the first time.

Chimpanzees are the only animal model for chronic HBV infection and have played a critical role in our understanding of HBV infection, as well as the development of vaccines and therapeutics [17,25,26]. The activity of MD ARC-520 + ETV was evaluated in nine chronically HBV-infected chimpanzees [17]. Iso-seq analysis from one HBeAg+ and one HBeAg− chimpanzee in that study demonstrated that 90% of HBsAg transcripts in the HBeAg+ chimpanzee were derived from cccDNA, whereas 66% of the HBsAg transcripts in the HBeAg− chimpanzee were derived from iDNA. The sequencing of serum HBV DNA demonstrated that there were no differences in the genome sequence contributing to the differential response nor was there selection against the siRNAs [17].

The deeper characterization of HBeAg+ and HBeAg− chimpanzee HBV transcripts by using Iso-seq in the current study provides a basis for comparison to the Heparc-2001 study patients’ HBV transcripts. This study includes an HBeAg-transitional chimpanzee that became HBeAg− during ETV treatment, allowing us the opportunity to assess how HBV transcripts changed during this process.

## 2. Materials and Methods

### 2.1. HBV siRNAs

All the siRNAs in these studies were cholesterol-conjugated and fully modified with 2′ fluoro or 2′-*O*-methyl to increase the stability of the siRNA and abrogate the innate immune response that would result from the use of RNA, as previously described [24]. ARC-520 comprised the Active Pharmaceutical Ingredient, two cholesterol-conjugated siRNAs named “siHBV-74” and “siHBV-77” at a 1:1 ratio, along with a delivery excipient named ARC-EX1 containing an *N*-acetylgalactosamine-conjugated melittin-like peptide (NAG-MLP) excipient that enhanced the delivery of the siRNAs into the cytoplasm of hepatocytes. The binding site for siHBV-74 was position 1779–1797, and the binding site for siHBV-77 was 1825–1843, relative to GenBank sequence V01460 that has DR1 at 1826–1836. The binding site for siRNA-75 was position 380–398, within the S coding sequence [24]. Cholesterol-conjugated siRNA was intravenously co-injected with a 1:1 weight/ weight quantity of ARC-EX1.

### 2.2. Chimpanzee Study

#### 2.2.1. Chimpanzee HBV Characterization

The initial study with nine chimpanzees aimed to determine the safety, tolerability, and pharmacological effects of ARC-520 on HBsAg-positive chimpanzees (*Pan troglodytes*) and was previously described [17,22]. HBV transcripts from the six chimpanzees identified in Table 1 are characterized in these analyses. As previously described, there are no records of experimental exposure to HBV for these animals [17]. Chimpanzees A2A004, A3A006, A4A014, and 95A010 had HBV-infected mothers and are thought to have become infected perinatally. Chimpanzees 88A010 and 89A008 were positive for HBsAg the first time that they were tested, which was at the ages of 6 and 15 years old, respectively. The HBV genomes of all the chimpanzees were sequenced from the liver DNA and RNA and determined to be of a chimpanzee HBV origin. The consensus sequence of HBV DNA in the liver was determined from liver biopsies collected after the ETV lead-in but prior to ARC-520 treatment day 1. Chimpanzee 95A010 was serotype adr, and the other five chimpanzees were serotype adw. Chimpanzee 88A010 had the basal core promoter variants A1762T/1764A, but all the others had wild-type sequences at the basal core promoter. None of these chimpanzees had the precore mutation.

#### 2.2.2. Chimpanzee Study Design

The chimpanzees received daily oral entecavir for an 8–20-week lead-in period prior to receiving intravenous ARC-520 (2–4 mg/kg) once every four weeks (Q4W) with continued daily ETV. After 6–10 doses of ARC-520, the siRNA siHBV-75 ± siHBV-74 were tested in the HBeAg− chimpanzees that responded with lower activity to ARC-520. The ETV dosing continued 1–2 weeks after the last siRNA dose. Plasma and serum were collected biweekly for the duration of the monitoring period to measure HBsAg, HBeAg, HBV DNA, anti-HBeAg, anti-HBsAg, clinical chemistries, and complete blood counts as previously described [17]. Periodic liver needle biopsies were collected to evaluate HBV DNA and RNA and for histology.

The study design had been to dose all the animals 12 times, but in anticipation of the chimpanzee reclassification by the U.S. Department of Fish and Wildlife, we ended the study ahead of schedule to monitor all the animals off treatment as long as allowed. All the sample collections from the chimpanzees were conducted prior to 14 September 2015, when the U.S. Department of Fish and Wildlife reclassified as endangered chimpanzees born in captivity at research centers.

### 2.3. Clinical Trial Heparc-2001 Extension Study (NCT 02065336)

This phase 1b study was a multidose open-label extension cohort of patients recruited by the Department of Medicine, Queen Mary Hospital, the University of Hong Kong through an amendment to the multi-center, randomized double-blind, placebo-controlled, dose-escalation study using an intravenous single-dose ARC-520 in combination with ETV in patients with CHB infection that had previously received a single injection of ARC-520. Upon completion of the multi-center study, all the patients enrolled in cohort 7 (6 HBeAg+ and 6 HBeAg− patients that were previously NA-naïve) were invited to participate in this extension study. Of these, 8 patients (3 HBeAg+ and 5 HBeAg−) consented to enroll into the extension study (denoted as cohort 10). The reason for the remaining 4 patients not participating in the extension study was related to the necessary time commitment. Both the initial multi-center study and the present single-center extension study were approved by the Institution Review Board (IRB) of the University of Hong Kong and Hospital Authority Hong Kong West Cluster, Hong Kong (UW 13-542). They were conducted in accordance with the 2008 Declaration of Helsinki and good clinical practice guidelines. All enrolled patients provided written informed consent for the study.

### 2.4. PacBio SMRT Sequencing and Analysis

#### 2.4.1. PacBio SMRT Sequencing

An Iso-seq library was prepared according to PacBio’s standard protocol. Briefly, cDNA was synthesized using the SMARTer PCR cDNA Synthesis Kit (Takeda, Mountain View, CA, USA) and was PCR-amplified. Following Blue Pippin size selection to create four size bins (<1 kb, 1–2 kb, 2–3 kb, and 3–6 kb), the cDNA was further amplified with PCR, ligated to hairpin adapters to create the SMRTbell Template, annealed to sequencing primer, and bound to polymerase for sequencing on the RSII sequencer (PacBio, Menlo Park, CA, USA via the generation of concatemers. For chimpanzee 88A010, the size bins were 1–2 kb, 2–3 kb, 3–6 kb, and >6 kb.

#### 2.4.2. HBV Reference Sequences and Genome Assembly

To obtain accurate full transcript alignment, it is necessary to construct a hybrid genome sequence containing >1× content. Based on the known start and end of HBV transcripts that are greater than one genome length, we concatenated the genome sequence from coordinates 2700–3182 + 1–3182 + 1–2100 (“2700–2100”) into a single genome fasta file. This was easily achieved by constructing the sequence in the DNA sequence editor SnapGene and then converting it to text using a quality text editor (VScode) because the final genome sequence was small (~5.7 kb). See the Supplementary Materials and Methods for more on chimpanzee reference sequences.

The reference genome for the alignment of human HBV transcripts of the Heparc-2001 patients was GenBank accession number AB073827 of genotype B/C that was 3215 bp. This genome sequence was concatenated from 2700–3215 + 1–3215 + 1–2200 (“2700–2200”) to accommodate transcripts that were longer after the HBV PAS.

After building these genomes, we empirically determined these concatemer genome sequences to be of an acceptable length by viewing the full-length non-concatemer (FLNC) alignment data in a genome browser (Jbrowse2). We found that our chosen concatemer length had perfectly aligning FLNC transcripts starting briefly after the start and ending ~50 bp from its end. Other concatenated genome sizes were also attempted (1600–3182 + 1–3182 + 1–2100, “1600–2100”), but they suffered from an early transcript start or late transcript termination.

#### 2.4.3. Full-Length Non-Concatemer (FLNC) Transcript Processing and FLNC Alignment

Subreads were collapsed to circular consensus sequence (CCS) reads using Pacbio’s internal CCS tool using the default parameters (https://github.com/PacificBiosciences/ccs (accessed on 6 December 2024)). Lima was used with the argument -peek-guess to discover the primer sequences and demultiplex CCS reads for individual samples (https://github.com/pacificbiosciences/barcoding/ (accessed on 6 December 2024)). FLNC transcripts were produced from demultiplexed reads using isoseq3 with the refine subcommand requiring poly-A sequences. Finally, FLNC transcripts were clustered into complete transcripts which were guided by the FLNC quality (https://github.com/PacificBiosciences/IsoSeq (accessed on 6 December 2024)). Following production, the FLNC reads were aligned, sorted, and indexed using PBMM2 (https://github.com/PacificBiosciences/pbmm2 (accessed on 6 December 2024)) with the default conditions for --preset “ISOSEQ”.

#### 2.4.4. Binning Reads into the HBV Transcriptome

We binned the Iso-seq read alignments into a pre-defined HBV transcriptome using a custom python program provided at https://github.com/Sandman2127/HBV_transcript_sequencing_analysis (accessed on 6 December 2024). Briefly, the program bins aligned the FLNC reads by parsing each read’s alignment start and CIGAR string from a SAM format file to determine the alignment locations and any breaks. Using the coordinates discovered the program searches for an appropriate transcript bin. To control for the differences in the sequencing library depth, the raw read counts were normalized via the reads per million (RPM) normalization technique. Finally, plots and tab separated value (.tsv) summary files of the transcript binning process were outputted.

Supplementary Table S2 shows the relative locations of the open reading frames, transcription start sites, and major splice sites for HBV genes in the natural genome (1×) and the 2700–2100 concatemer genome of chimpanzees and the 1× and 2700–2200 concatemer genome of AB073827 for the human patients.

## 3. Results

### 3.1. Differential Changes in the HBV Parameters During the siRNA Study in HBeAg-Positive and HBeAg-Negative Chimpanzees

**Figure 2:**
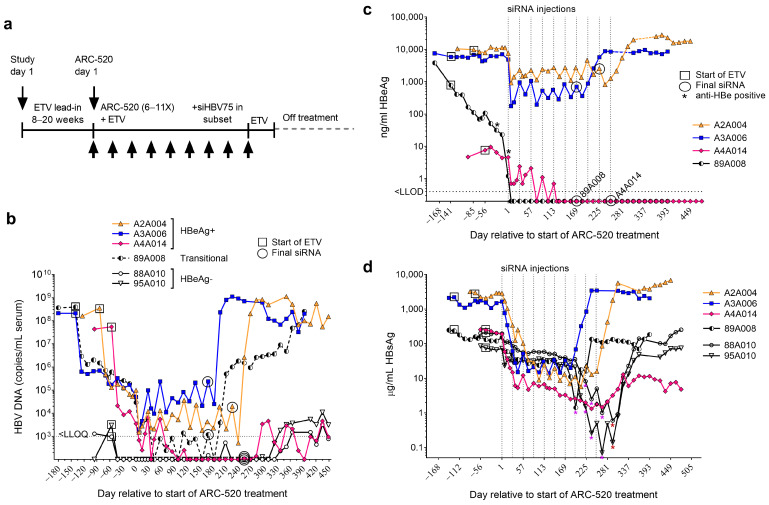
Response to multi-dosing (MD) of chimpanzees with HBV siRNAs. (**a**) Chimpanzees received daily entecavir (ETV) beginning on study day 1 for an ETV lead-in period of 8–20 weeks, followed by MD with 6–11 intravenous injections of ARC-520 beginning on ARC-520 day 1 with continued ETV treatment. Some chimpanzees received injections of siRNA siHBV-75 (HBsAg nadir following dose indicated by purple asterisk) or siHBV-75 + siHBV-74 (HBsAg nadir indicated by dark red asterisk) after ARC-520 MD. Black asterisk (**c**) indicates when animals became anti-HBeAg positive. ETV treatment continued 1–2 weeks after the final siRNA dose. (**b**–**d**) HBeAg-positive, HBeAg-transitional, and HBeAg-negative chimpanzees had a pre-study health check (HC) 40 days prior to study day 1, at which time, the ETV lead-in (box) began prior to commencing Q4W dosing with ARC-520, beginning on ARC-520 day 1. Timing of the final siRNA injection, either ARC-520 or siHBV-75 ± siHBV-74 + ARC-EX1 is indicated by a circle. Serum HBV DNA (**b**), HBeAg (**c**), and HBsAg (**d**) measurements are shown relative to day 1 of ARC-520 treatment.

The HBV transcripts in the liver were characterized from six chimpanzees in a multiple dosing (MD) study of HBV siRNA administered Q4W along with daily oral ETV and a follow-up period off all treatment (Figure 2a). Four of these chimpanzees were HBeAg+, and two were HBeAg− at the pre-study health check (HC). As previously reported, the HBeAg+ chimpanzees responded to the ETV treatment with a 4.0 ± 0.2 log_10_ copies/mL mean decline in serum HBV DNA during the ETV lead-in, whereas the HBeAg− chimpanzees had pre-treatment serum HBV DNA levels close to the lower limit of detection (LLOD) that became undetectable after two weeks of the ETV treatment (Figure 2b). Chimpanzee 89A008 became HBeAg− during the ETV lead-in, prior to the siRNA treatment, identifying this animal as “transitional” for HBeAg (Figure 2c).

After a lead-in period of 8–20 weeks with ETV to reduce the viral replication prior to the siRNA treatment, the chimpanzees received 6 to 10 injections Q4W of ARC-520 in conjunction with continued daily oral ETV (Table 1). Excluding the transitional chimpanzee, HBsAg was reduced by 0.9 to 1.4 log_10_ in the HBeAg+ animals and by 0.4 to 0.7 log_10_ in the HBeAg− animals following the first dose of ARC-520, as previously reported (Figure 2d, [17]). Following ARC-520 MD, the HBeAg− chimpanzees received an siRNA (siHBV-75) that targeted HBV RNA in the S gene coding region, which is upstream of the DR2-DR1 region (Table 1). As shown in Figure 2d, HBeAg− chimpanzees 88A010 and 95A010 received three doses of siHBV-75, yielding deep reductions in HBsAg up to a 3.0 log_10_ decrease relative to study day 1, as previously reported. The continuation of this study and post-treatment results are reported here for the first time. After dosing with siRNA-75, these two chimpanzees were then given one dose of a combination of siHBV-75 plus siHBV-74, to target all the cccDNA-derived transcripts including X, which also yielded deep reductions in HBsAg (Figure 2d). A single dose of siHBV-75 given to transitional chimpanzee 89A008 after six doses of ARC-520 reduced HBsAg by 2.0 log_10_, relative to the maximum decrease of 0.9 log_10_ from ARC-520 alone. This result suggested transitional chimpanzee 89A008 produced a significant amount of HBsAg from iDNA at this time point, study day 337 (day 197 relative to the start of ARC-520 dosing).

The chimpanzees’ HBV liver DNA was measured via quantitative PCR with or without Plasmid Safe DNase (PSD) digestion, which digests dslDNA, fragments of iDNA, and to some degree rcDNA (Supplementary Table S1). This method was previously used for measuring cccDNA, though a focus group recently determined that PSD does not efficiently reduce rcDNA and improved the protocol [27].

The total liver HBV DNA in HBeAg+ chimpanzees A2A004, A3A006, and A4A014 decreased by 1.1–1.2 log_10_ copies/µg DNA following 8 to 20 weeks of ETV treatment and decreased further by 0.4–1.1 log_10_ with ARC-520 + ETV treatment. During the ETV or ETV + ARC-520 treatment period, the amount of liver HBV DNA was similar with or without DNase digestion, suggesting that the HBV DNA in these HBeAg+ animals was primarily cccDNA with little iDNA. During the ETV lead-in, cccDNA decreased by 0.1 to 0.3 log_10_ copies/µg. It decreased additionally by 0.3 to 0.7 log_10_ with ARC-520 + ETV treatment in the absence of HBeAg seroconversion. When off ETV, replication resumed and the total liver HBV DNA in HBeAg+ chimpanzees increased to the pre-treatment levels or higher (~7 log_10_ copies/µg) except in chimpanzee A4A014 that was able to control replication off all treatment (see Figure 2b).

Chimpanzee A4A014 was HBeAg+ pre-treatment, but her HBeAg was only 4.7 ng/mL, and HBsAg was one order of magnitude lower than the other two HBeAg+ chimpanzees (Table 2). Her alanine aminotransferase (ALT) prior to treatment was modestly elevated (<2× ULN). The low HBeAg along with the lack of any precore mutations in her HBV DNA [17] suggest that this chimpanzee pre-study was in the process of gaining immune control. The total liver HBV DNA in chimpanzee A4A014 declined from the pre-study to the end of the study by 2.0 log_10_ copies/µg. Like the other HBeAg+ chimpanzees, the liver HBV DNA quantities were similar with or without PSD digestion while on the ETV treatment, suggesting that this animal had primarily cccDNA. A cccDNA decrease of 0.5 log_10_ was associated with the ETV lead-in plus HBeAg seroconversion, and an additional 1.0 log_10_ decrease occurred during ARC-520 + ETV treatment. Only 3.7 log_10_ copies/µg HBV cccDNA remained in the liver on study day 379 off all treatment, equivalent to 0.03 copies/cell. In contrast, the other two HBeAg+ chimpanzees that were both young males had >6 log_10_ copies/µg +PSD HBV DNA at day 379 (>6 copies/cell).

Transitional chimpanzee 89A008 seroconverted for HBeAg during the 20-week ETV lead-in, decreasing from 3797 ng/mL HBeAg pre-treatment to 1.2 ng/mL HBeAg just prior to the first ARC-520 dose, and his HBsAg expression pre-study was nearly the same as that of chimpanzee A4A014 that became HBeAg− on ETV + ARC-520 treatment (Table 1). His ALT, however, was 3×–5× ULN during the HBeAg seroconversion. The total liver HBV DNA in chimpanzee 89A008 decreased 2.5 log_10_ copies/µg during the ETV lead-in and HBeAg-seroconversion while the +PSD liver DNA decreased by 1.3 log_10_ copies/µg during this period. Unlike the other HBeAg+ chimpanzees, however, the +PSD HBV liver DNA was consistently less than the undigested total HBV DNA (4- to 19-fold less) during ETV treatment, suggesting that he had more iDNA than the HBeAg+ chimpanzees. Chimpanzee 89A008 was 24 years old, whereas the HBeAg+ chimpanzees were 9–12 years old at the start of the study (Table 1).

At the final liver DNA analysis on day 379, the now HBeAg− chimpanzees 89A008 and A4A014 had a similar amount of +PSD HBV liver DNA (3.7 log_10_ copies/µg). However, the study outcomes post-treatment differed for these two animals. A4A014 controlled replication with serum HBV DNA fluctuating around the lower limit of quantitation (LLOQ), as serum DNA did for the HBeAg− chimpanzees 88A010 and 95A010 (Figure 2b), and ALT normalized. She had transitioned to HBeAg− chronic infection, previously known as an inactive carrier state. Chimpanzee 89A008, on the other hand, had serum HBV DNA that was suppressed for about four months, but thereafter fully returned to the baseline (>8 log_10_ copies/mL serum). Although his ALT normalized during ARC-520 + ETV treatment, ALT increased post-treatment to approximately 3× ULN off all treatment. This chimpanzee had transitioned to HBeAg− chronic hepatitis B.

For HBeAg− chimpanzees 88A010 and 95A010, the total liver HBV DNA decreased minimally during the ETV lead-in and by ≤0.7 log_10_ copies/µg during siRNA + ETV treatment. The +PSD DNA decreased by ≤0.6 log_10_ copies/µg. The decreases were during siHBV-75 + ETV treatment. For both HBeAg− animals, the +PSD liver DNA had fewer copies of HBV than the undigested samples, pointing to iDNA as a component of the total HBV DNA. Although orders of magnitude lower than in the HBeAg+ chimpanzees, these HBeAg− animals had cccDNA, as evidenced by their periodic replication of HBV near the LLOQ (Figure 2b). The day 379 quantities of +PSD HBV liver DNA were 2.54 and 3.28 log_10_ copies/µg for chimpanzees 88A010 and 95A010, equivalent to 0.002 and 0.01 copies/cell, respectively. The more similar quantities of liver HBV DNA with or without PSD in chimpanzee 95A010 (18-year-old female), relative to this difference in chimpanzee 88A010 (25-year-old male), suggest that she had less iDNA than the older male.

At the end of the study off all treatment, HBeAg− female chimpanzees 95A010 and A4A014 both had low cccDNA, low iDNA, low HBsAg, normal ALT, and controlled replication with cyclical increases and decreases in serum HBV DNA. Three initially high-viremia males (A2A004, A3A006, and HBeAg-transitional 89A008) returned to their high viremia state in the absence of ETV, despite 89A008 becoming HBeAg− with lower HBsAg and at least transiently less cccDNA post-treatment than the other two. The liver DNA was not measured at the very end of the study period (study day 533).

Our previous results characterized some of the differences between HBeAg+ and HBeAg− chimpanzees that suggested HBeAg− chimpanzees had fewer products of cccDNA and more HBV integrated into the host DNA. These results are included to provide the background for further transcript analysis of the HBeAg+, HBeAg−, and HBeAg-transitional chimpanzees. The number of precore/core transcripts, measured with a quantitative reverse transcription PCR (RT-qPCR) probe that annealed in the precore/core coding sequence, comprised only 3.5 ± 0.5% of the total transcripts in the HBeAg− chimpanzees, whereas they comprised 52.6 ± 0.5% of the total transcripts in the HBeAg+ chimpanzees [17]. The alignment of the full-length Iso-seq transcripts to the HBV genome revealed that 90.5% of the S transcripts from HBeAg+ chimpanzee A2A004 had the expected 5′ start sites and 3′ ends at the HBV PAS. In contrast, most of the S transcripts of HBeAg− chimpanzee 88A010 aligned with the S promoters but failed to align with HBV beginning between DR2 and DR1 to the HBV PAS. Most of chimpanzee 88A010’s transcript sequences were fused to chimpanzee host sequences (66.4%), an HBV sequence (10.1%), or a sequence of unknown origin (0.9%) and terminated at cryptic polyadenylation signals; and 32.8% of the transcripts terminated following an alternative PAS located 122 bases upstream of the HBV PAS [17]. Far fewer (0.3%) of the transcripts in HBeAg+ chimpanzee A2A004 terminated at the alternative PAS, supporting the termination at this alternative PAS being a feature of iDNA transcription. A similar difference was noted between HBeAg+ chimpanzee A3A006 and HBeAg− chimpanzee 95A010 [22].

### 3.2. Realignment of HBV Genome to Visualize Unconventional Transcripts

Our prior Iso-seq analysis was focused on understanding the differences in the HBsAg response to ARC-520 in chimpanzees, which uncovered the observation that an abundant source of HBsAg was iDNA. Those samples were all collected after the ETV lead-in. The current study is an in-depth analysis of all the transcripts that align to HBV from chimpanzee liver biopsies collected at multiple time points, on or off ETV, as shown in Table 2.

**Figure 3:**
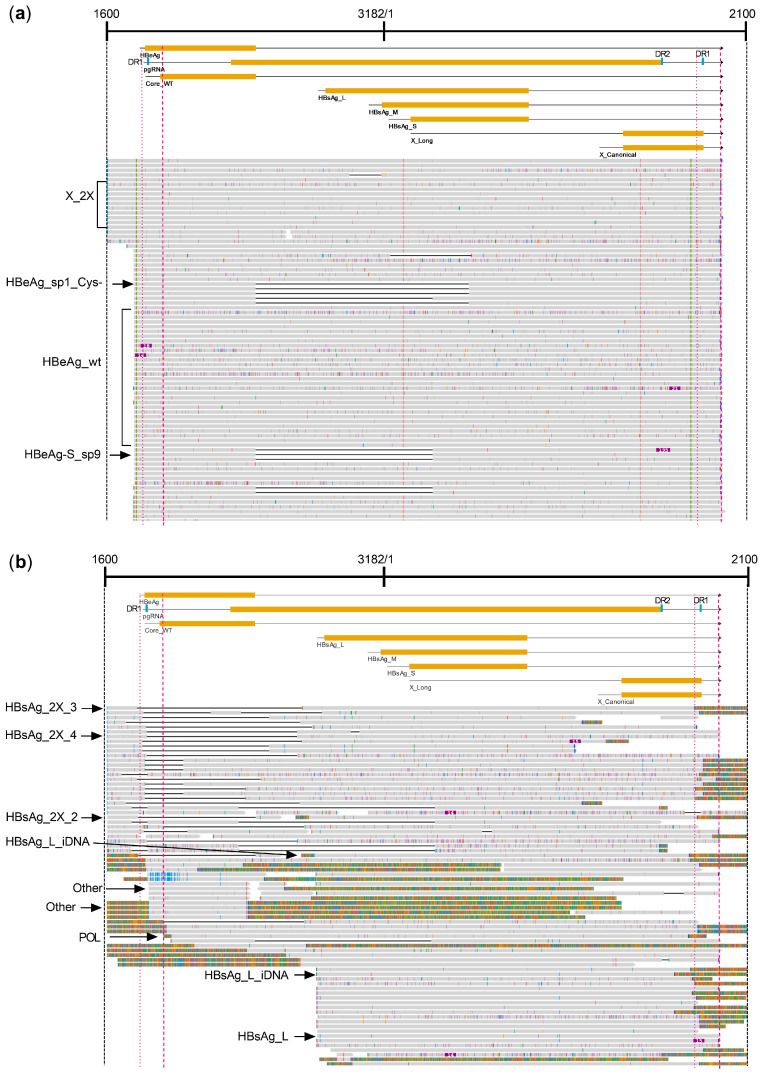
Alignment of chimpanzee HBV transcripts to chimpanzee HBV genome 1600–2100 concatemer. Alignments of just the longest transcripts of HBeAg+ chimpanzee A2A004 on study day 85 (**a**) and HBeAg− chimpanzee 88A010 on study day 57 (**b**), in both cases following the ETV lead-in and prior to the first dose of ARC-520. Arrows point to characteristic transcripts in these samples. Labels of the indicated characteristic transcripts are listed and explained in Table 3. Solid gray lines represent sequences perfectly aligned to the HBV genome concatemer of chimpanzee 88A010; black lines represent spliced-out sequences or large deletions; purple marks indicate base insertions; small gray marks indicate single-base deletions; individual base changes are green for adenine, orange for guanine, red for thymine, and blue for cytosine; multi-color lines indicate sequences not aligning to the HBV genome concatemer; dotted pink lines indicate the alternative PAS (30 bases upstream of DR1); and dashed pink lines indicate the HBV PAS.

The greater than genome length precore (HBeAg) transcript and pgRNA and all the other expected S and X transcripts can be aligned to the 1600–2100 HBV genome, as shown for the HBeAg+ chimpanzee A4A014 in Supplementary Figure S1A. We observed, however, that dozens of transcripts started upstream of position 1600 and extended past the first HBV PAS; and those in the HBeAg+ chimpanzees differed from those in the HBeAg− chimpanzees (the alignments of the longest transcripts from the representative HBeAg+ and HBeAg− chimpanzees are shown in Figure 3).

**Figure 4:**
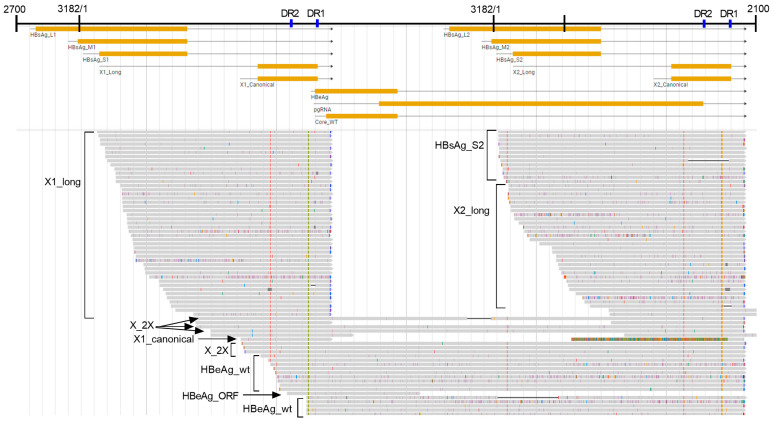
Alignment of HBeAg+ chimpanzee A2A004_d85 transcripts to HBV genome concatemer 2700–2100. The schematic shows positions of transcripts with ORFs as yellow bars. Arrows at the ends indicate the HBV PAS. Solid gray lines represent sequences perfectly aligned to the HBV genome concatemer of chimpanzee 88A010; black lines represent spliced-out sequences or deletions; purple marks indicate base insertions; small gray marks indicate single-base deletions; individual base changes are green for adenine, orange for guanine, red for thymine, and blue for cytosine; the multi-color line indicates sequence not aligning to the HBV genome concatemer. Labels of the indicated characteristic transcripts are listed and explained in Table 3. Examples of the transcripts X1_long, X_2X, X1_canonical, HBeAg_wt, HBeAg_ORF, HBsAg_S2 and X2_long are indicated.

To capture these unconventional transcripts, we aligned them to a 2700–2100 genome concatemer (Supplementary Figure S1B). This template allowed the alignment of S and X transcripts in two locations and a single alignment for pgRNA and precore, but the transcript binning program only allowed each transcript to be counted at one of the two genome locations. Supplementary Table S2 and Methods define the start sites, ORF positions, and major splice sites for HBV gene products on the natural genome (1×) and the 2700–2100 concatemer genome. This extended template allowed the alignment of S and X transcripts that started prior to 1600 and extended beyond the first HBV PAS up to the second PAS (Figure 4).

Each HBV transcript was identified first by reference to the gene product it had the potential to encode, based on the nearest open reading frame known to be an HBV gene after a defined start site for the 5′ untranslated region (5′ UTR). Studies of 5′ UTRs have determined that the average length of the 5′ UTR in humans is 210 bases, the minimum length is 18 bases, and the maximum length is 2803 bases [28]. The minimum length for all vertebrates is between 15 and 20 bases. Our definitions allowed a minimum of 15–20 nt for the 5′UTR. A minimum of 20 nt was required for HBeAg, core, and M-HBsAg because transcripts with a 5′ UTR less than this would more likely be translated as core, pol, and S-HBsAg, respectively.

After identifying the HBV protein product likely to be encoded in the transcript, additional distinguishing features were identified. The individual characteristics of gene products included the 3′ termination site, whether at the HBV PAS or prior to the PAS, and splice sites. For example, four transcript types could potentially encode HBeAg with a wild-type sequence, and one product (HBeAg_sp1_Cys−) would encode HBeAg lacking the terminal cysteine due to having the sp1 splice sites (Table 3). The start sites, open reading frame, and splice sites for each transcript are identified in Supplementary Table S2 and in the code (https://github.com/Sandman2127/HBV_transcript_sequencing_analysis/tree/main/HBV_transcript_data (accessed on 17 December 2024)). Gene product names and definitions, transcript counts, and percentages within the combined HBeAg+ or HBeAg− chimpanzee HBV transcriptomes are shown in Table 3 (using the HBeAg status from Table 2).

**Figure 5:**
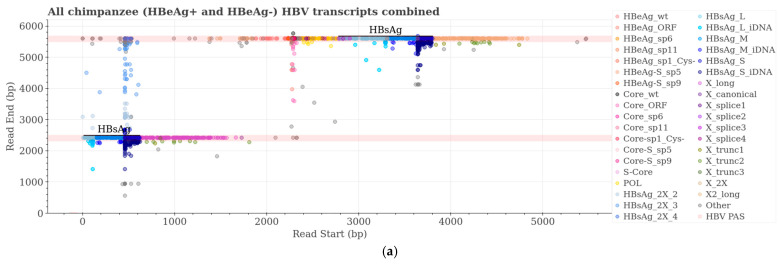

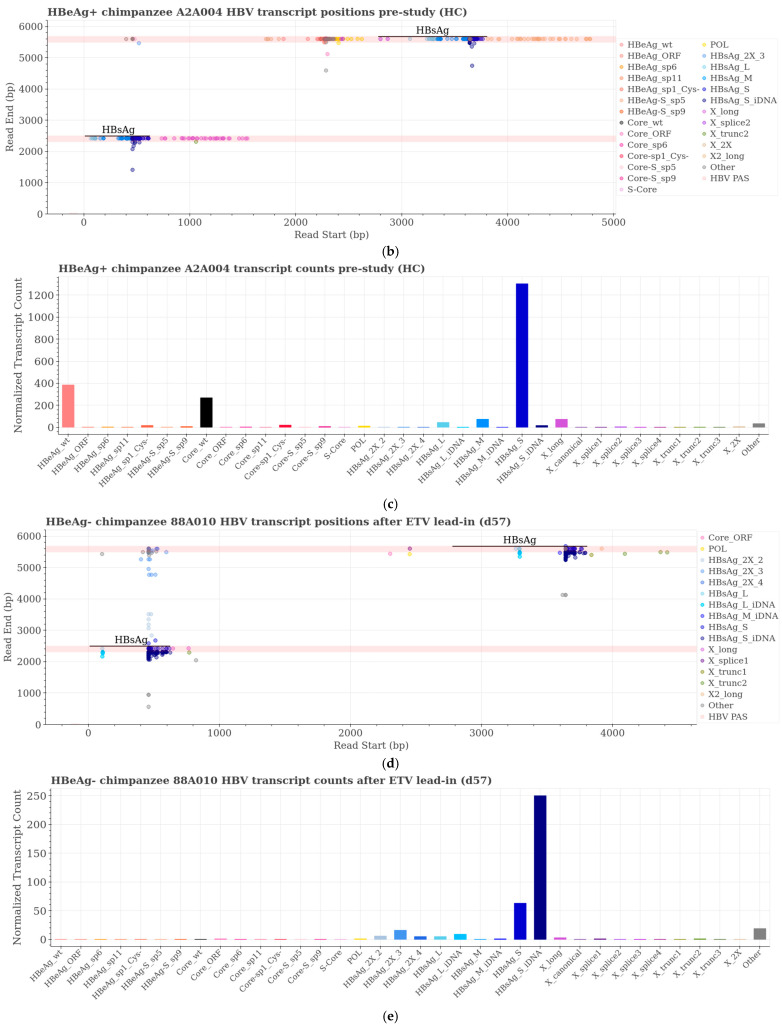

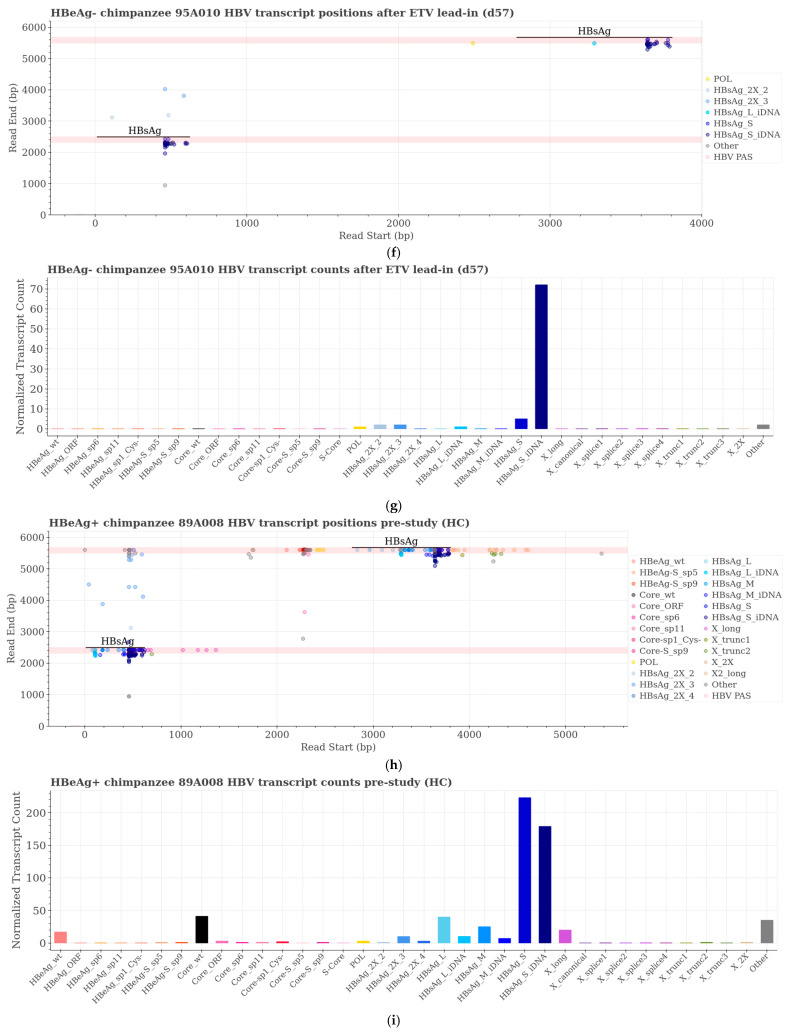

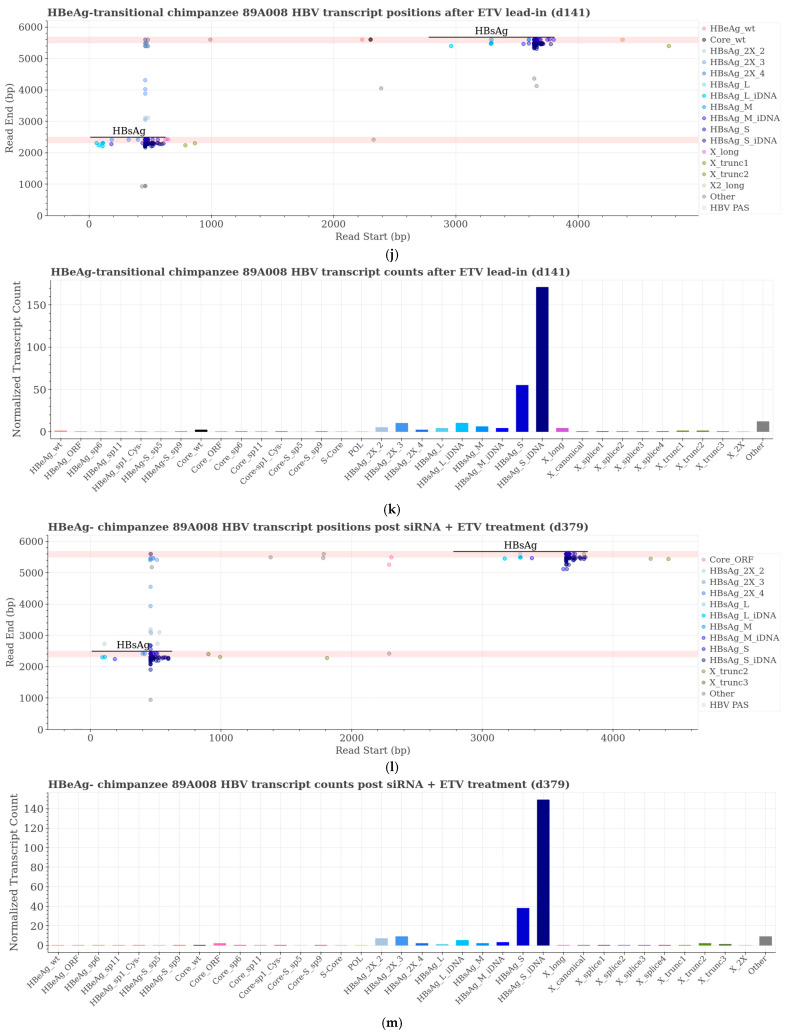
Chimpanzee HBV transcripts aligned by read start and read end positions on 2700–2100 concatemer and normalized transcript counts by sample. Identified HBV transcripts aligned to the 2700–2100 concatemer are from all chimpanzee samples (**a**), HBeAg+ chimpanzee A2A004 at pre-study HC (**b**,**c**), HBeAg− chimpanzees 88A010 (**d**,**e**) and 95A010 (**f**,**g**) after ETV lead-in day 57, and HBeAg-transitional chimpanzee 89A008 at HC (**h**,**i**), after ETV lead-in on day 141 (**j**,**k**) and at day 379 off treatment (**l**,**m**). Animal ID, HBeAg-status, and time point are in the title of each graph. Pink bars, HBV PAS. Descriptions of transcripts are in Table 3.

The HBV transcripts from all the chimpanzee samples together were graphed by the starting and ending points of each transcript to visualize the variety of transcripts on a “position and type” graph (Figure 5a). The pink bars across each graph indicate the location of the HBV PAS (two on the 2700–2100 concatemer). For the animal and time point, the graphs of normalized transcript counts follow the “position and type” graphs (Figure 5). As expected, most of the transcripts in the HBeAg+ chimpanzees terminate on the pink bars, indicating cccDNA-derived transcripts (Figure 5b and Supplementary Figure S2). Most of the transcripts from HBeAg− chimpanzees 88A010 and 95A010 fall below the PAS bars and are described with the term “iDNA” (Figure 5d,f). The types of transcripts shift for HBeAg-transitional chimpanzee 89A008 as he became HBeAg−, from the inclusion of transcripts terminating at the HBV PAS (cccDNA-derived) at HC, with less of these after the ETV lead-in on day 141, to having very few cccDNA-derived transcripts after treatment on day 379 (Figure 5h–m). Even at the HC, this chimpanzee had many HBV transcripts that terminated prior to the HBV PAS (iDNA-derived).

The transcript counts and percentage of each are shown by chimpanzee and time point in Supplementary Table S3. All transcript sequences are listed in Supplementary Table S4.

### 3.3. HBV Transcripts with the Potential to Encode HBx in Chimpanzees

Four of the five categories of X transcripts that encode the entire X ORF and have the potential to produce HBx protein are canonical X (X_canonical), X mRNA with a long 5′ UTR (X_long), an X transcript with a complete ORF but terminating prior to the HBV PAS (X_trunc3), and a long transcript encoding two copies of the X ORF that terminates following the second HBV PAS (identified as gene product X_2X). Out of thirteen liver biopsies collected from six chronically HBV-infected chimpanzees that had a total of 12,396 normalized transcripts aligning with HBV, only a single transcript was identified as canonical X with a start site between 1240 and 1361 and terminating after the HBV PAS (Figure 4 and Figure 5a, and Table 3). However, 31 transcripts of the normalized chimpanzee samples were X_2X, 0.27% of the total HBeAg+ chimpanzee HBV transcriptomes but 8.2% of the transcripts encoding the X ORF, and several of these started at the canonical X start site.

The raw read HBV transcriptomes had a total of 35 transcripts identified as X_2X (Supplementary Table S4). Upon inspection of the sequenced transcripts, only 5/35 (14%) had the same X gene sequence in both locations within the transcript. Supplementary Figure S3 shows one of these X_2X sequences in which the first X gene had a 5′ UTR-like that of canonical X and had an ORF that would encode the HBx protein, but the second X gene had an insertion that shifted the reading frame. The transcripts in which both X ORFs were identical may represent transcription that by-passed the HBV PAS upon the first encounter and terminated at the same sequence following the second encounter, as has previously been suggested [29]. In most cases, however, both X gene locations had numerous mutations that would prevent the expression of HBx, and those in the first position differed from those in the second. This observation argues against transcription around a 1X genome and instead suggests the transcription of ligated dslDNA dimers.

**Figure 6:**
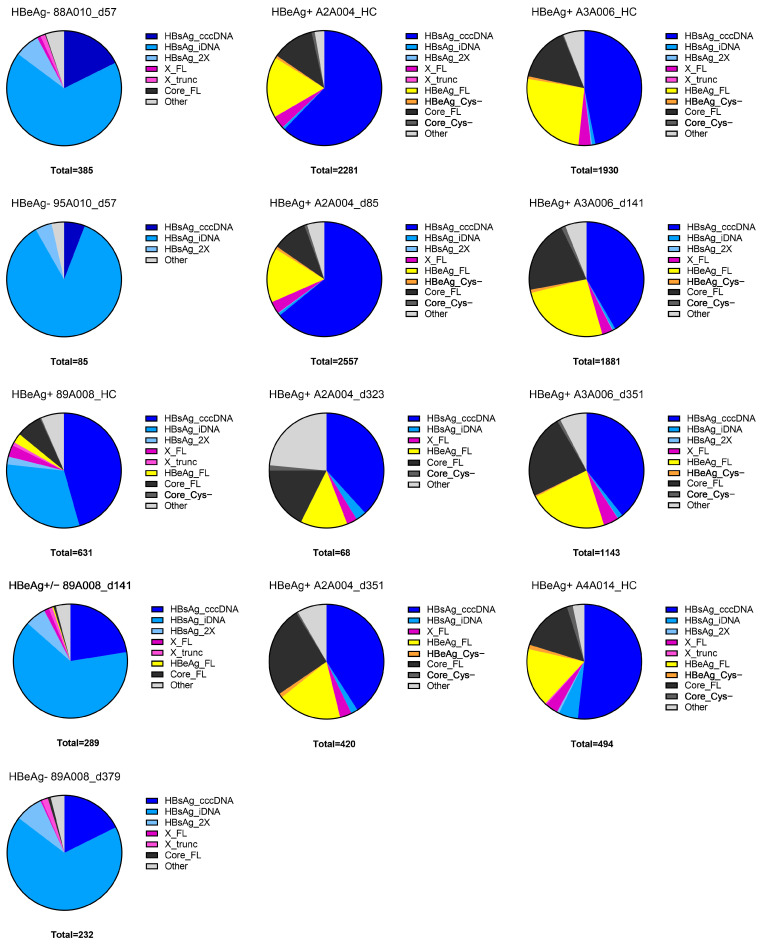
Major HBsAg, HBeAg, core, and X transcript counts in each chimpanzee sample. Major HBsAg transcripts are HBsAg_cccDNA, HBsAg_iDNA, and HBsAg_ 2X; X transcripts are full-length X (X_FL) and truncated X (X_trunc); HBeAg transcripts are full-length (HBeAg_FL) or HBeAg_Cys−; core transcripts are full-length (Core_FL) or Core_Cys−; “Other” here includes the transcripts not specifically identified as well as the fusion transcripts (HBeAg-S, Core-S, and S-Core). Chimpanzee identification, time point, and HBeAg status are indicated.

The major transcript (91.0%) with the potential to encode full-length HBx in HBeAg+ chimpanzees is long X with its variable transcription start sites that begin distal to the S-HBsAg start site and proximal to the canonical X start sites and terminate at the HBV PAS. An X transcript with a complete ORF but terminating prior to the HBV PAS (X_trunc3) comprised only 0.8% of all transcripts that theoretically could produce full-length HBx protein in chimpanzees (Supplementary Table S3). All these transcripts with the potential to produce full-length HBx protein comprised approximately 3% of the HBV transcriptome in the HBeAg+ chimpanzees but ≤1% in the HBeAg− chimpanzees (Figure 6 and Supplementary Table S5).

### 3.4. HBV Transcripts with the Potential to Encode HBsAg in Chimpanzees

HBV transcripts with the L-, M-, or S-HBsAg ORFs that have a polyadenylated 3′ terminus are expected to encode HBsAg, whether they have the HBV PAS or a cryptic PAS from an integration product. We are defining HBsAg transcripts with an HBV sequence that terminates anywhere from immediately distal to the stop codon up to the proximal end of the HBV PAS as being from iDNA (HBsAg_iDNA) and HBsAg transcripts that terminate distal to the HBV PAS as being from cccDNA (HBsAg_cccDNA). These products are easily distinguished on the position and type graphs in Figure 5 and Supplementary Figure S2 because those that end with the HBV PAS appear as circles on top of the pink HBV PAS bars across the graph, and the iDNA products are below the PAS bars. More than 92% of all transcripts in the HBeAg− chimpanzees encoded HBsAg, whereas 41–65% of the transcripts in the HBeAg+ chimpanzees encoded HBsAg (Figure 6 and Supplementary Table S5). HBsAg_iDNA transcripts were 67.5–85.9% of the transcripts in the HBeAg− chimpanzee samples but 0.7–5.5% of the transcripts in the HBeAg+ chimpanzee samples; the highest percentage of the latter (5.5%) was in chimpanzee A4A014 that became HBeAg− during MD ARC-520 plus ETV treatment.

HBsAg transcripts which aligned to the 2700–2100 genome identified a novel transcript category that we named HBsAg_2X (Table 3). These transcripts encode HBsAg but terminate distal to a deleted HBV PAS, as though the template were a cccDNA with the HBV PAS deleted (HBsAg_2X_2 and HBsAg_2X_3). In some cases, the transcript terminated at a distant HBV PAS as though transcribed from two tandem genomes, the first lacking the PAS and the second including it (category HBsAg_2X_4). Some of these transcripts comprise only an HBV sequence, and some are terminally fused to host sequences. Examples of each of these products can be seen in the upper transcript alignments from chimpanzee 88A010 as shown in Figure 3b, although the 1600–2100 alignment template does not show the upstream HBsAg coding sequence. The theoretical category HBsAg_2X_1 was defined as an S transcript with a large deletion after the S ORF that terminated within 300 bases distal to the HBV PAS; however, such transcripts were not observed.

The HBsAg_2X transcripts can be visualized well on the “position and type” graph (Figure 5a) because transcript reads start around position 460, and reads end between 2500 and 5600, falling between the upper and lower HBV PAS bars. HBsAg_2X transcripts were uncommon in the HBeAg+ chimpanzee samples but comprised 7.0% and 4.7% of the transcripts of HBeAg− chimpanzees 88A010 and 95A010, respectively (Figure 5 and Supplementary Table S5). 2.2% of transitional chimpanzee 89A008’s transcripts were HBsAg_2X pre-study (HC), and this percentage increased to 7.8% by study day 379 when the chimpanzee was off all treatment (Supplementary Table S5). These transcripts are visualized and quantified in Figure 5h–m.

### 3.5. HBV Transcripts with the Potential to Encode HBeAg, Core, or Polymerase

Full-length HBeAg protein can be produced by the wild-type (wt) precore mRNA (HBeAg_wt), or by precore transcripts that terminate after the HBeAg ORF prior to the HBV PAS (HBeAg_ORF), or from the sp6 or sp11 splice products of precore that have a splice site distal to the stop codon (Table 3). In the combined HBeAg+ chimpanzee HBV transcripts, 18.6% were wt precore, and each of the other three categories that could encode precore comprised only 0.3% of the total. Pre-study, these precore transcripts comprised 16.6% to 25.9% of the HBeAg+ chimpanzees’ HBV transcriptomes and 2.7% of the HBeAg-transitional chimpanzee’s HBV transcriptome (Figure 6 and Supplementary Table S6). The sp1 splice product of precore terminates without the terminal cysteine residue (HBeAg_Cys−). It comprised 0.7% of the combined transcriptome and was detected only in the HBeAg+ chimpanzee samples.

Similarly, core can be produced from pgRNA (core_wt), core_ORF, and from the sp6 or sp11 splice products of pgRNA. The percentages of pre-study transcripts with the potential to encode core varied from 11.9% to 15.5% in the HBeAg+ chimpanzees (Figure 6 and Supplementary Table S6). Interestingly, this percentage increased to 25.7% and 23.7% in HBeAg+ chimpanzees A2A004 and A3A006, respectively, on study day 351, at which point, viremia had returned to the pre-study levels, but HBsAg was still suppressed. Precore transcripts, however, did not increase. Study day 351 was six weeks after the last ARC-520 injection and 33 days after the last dose of ETV.

PgRNA transcripts spliced as sp1 encode HBV core protein that lacks the terminal cysteine residue, a product that plays a role in enhancing replication [30]. These comprised 0.3% to 1.6% of the HBV transcriptome in the HBeAg+ chimpanzees but were not detected in the HBeAg− chimpanzees.

Polymerase could be translated from pgRNA or a transcript starting distal to DR1 but prior to the translation start site and terminating either at or prior to the HBV PAS (“POL”). Both the HBeAg+ and HBeAg− chimpanzees had some of these POL transcripts, but in the HBeAg+ chimpanzees, they terminated mostly at the HBV PAS, and in the HBeAg− chimpanzees, they terminated prior to the HBV PAS. POL transcripts were only 0.7% of all the HBV transcripts.

Neoantigens are expected to result from the many insertions, deletions, and quasispecies. These are discussed in the Supplementary Materials.

### 3.6. Transition from HBeAg-Positive to HBeAg-Negative

**Figure 7:**
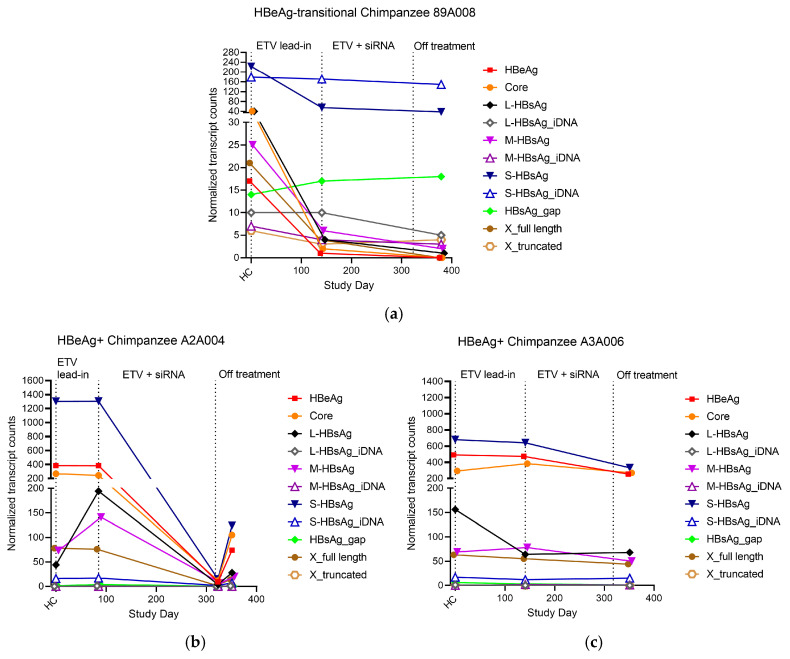
Change in HBV transcripts during transition from HBeAg+ to HBeAg− in chimpanzee 89A008. (**a**) Chimpanzee 89A008 was HBeAg+ at HC, became HBeAg− during the ETV lead-in that ended on day 141, and remained HBeAg− off treatment on day 379. Transcripts in HBeAg+ chimpanzees A2A004 (**b**) and A3A006 (**c**) were measured at HC, after ETV lead-in, one week off treatment (d323) in A2A004, and off treatment on day 351.

In the HBeAg-transitional chimpanzee, 45.6% of its S transcripts were from cccDNA at the pre-study health check, and this percentage declined to 22.5% after the ETV lead-in, further declining to 17.7% by study day 379 off all treatment (Figure 6 and Supplementary Table S6). At the same time points, the percentages of S transcripts from iDNA increased from 31.1% to 67.7%. An evaluation of the number of transcripts before and after ETV lead-in demonstrated that the cccDNA-derived transcripts all decreased as this chimpanzee became HBeAg-negative, while the iDNA transcripts remained similar (Figure 5h–m and Figure 7a). By contrast, the cccDNA-derived transcripts in the two HBeAg+ chimpanzees remained similar at comparable time points (Figure 7b,c). RNAseq analysis previously demonstrated that the transcripts of these chimpanzees were deeply reduced (>90%) by ARC-520 [17]. These decreases are not shown here except that the expression on day 323 for chimpanzee A2A004 had not yet rebounded.

### 3.7. Characterization of HBV Transcripts in Patients That Had Previously Been Treated with ARC-520

The HBeAg+ and HBeAg− patients in the study Heparc-2001 cohort 10 received a single dose (SD) of ARC-520 and then 32–40 weeks later received four to nine doses given Q4W (MD), followed by a period of 20–23 months of ETV only [31]. Demographics, treatment details and viral parameters for the eight patients in cohort 10 including values at the baseline, at treatment nadir, and at the last follow-up have been published (Supplementary Table S7 [31]). Six patients consented to a liver biopsy at a follow-up visit 19.2 to 23.6 months after the last dose of ARC-520, and five of these had sufficient material for an intrahepatic virologic profile, immunohistochemical staining (Supplementary Table S8), and for Iso-seq HBV transcript analysis, the subject of this study (Table 4).

At the time of the biopsy, the serum HBV DNA was undetectable or <LLOQ in all five patients who had biopsies. All these patients except patient 709 remained on ETV. The total transcript counts and quality of patient Iso-seq samples was as good as for the chimpanzee samples (Supplementary Table S8), but no HBV transcripts were detected in HBeAg− patients 701 and 709. As previously reported, patient 709 cleared HBsAg following MD ARC-520, developed antibodies to HBsAg, and had no detected HBsAg-positive hepatocytes during histological evaluation (Supplementary Tables S7 and S8). No HBV markers were detected in his serum at follow-up. Patient 701 had undetectable serum HBV DNA, HBV RNA, and HBcrAg at the last follow-up with only 1.3 IU/mL HBsAg. Patients 701 and 709 had lower amounts of cccDNA than the other patients, i.e., 0.382 and 0.263 copies/cell, respectively. In both cases, their total liver HBV DNA was about 0.9 copies/cell, suggesting that they had more integrated HBV than cccDNA.

As previously reported, the other two HBeAg− patients, patients 705 and 712, had 1.0 and 1.1 copies/cell cccDNA at follow-up, respectively (Supplementary Table S8). Patient 705 had more than twice the amount of total HBV DNA in the liver (4.4 copies/cell) than patient 712 (1.8 copies/cell). The alignments of Iso-seq HBV transcripts to the HBV genome are shown in Supplementary Figure S4. A total of 6.3% and 0% of all transcripts were cccDNA-derived in patients 705 and 712, respectively (Supplementary Table S9). Patient 705 had almost twice as many Iso-seq transcripts as patient 712: 127 and 66, respectively (Table 4). These transcript counts in both cases are similar to those of HBeAg− chimpanzee 95A010. Like chimpanzees, these patients’ S transcripts were primarily from iDNA: 21.3% and 19.7% of the total HBV transcripts were HBsAg from iDNA whereas only 2.4% and 0% were cccDNA-derived S transcripts (Table 4). In both patients, 40% of their hepatocytes were previously shown to be HBsAg-positive (Supplementary Table S8).

**Figure 8:**
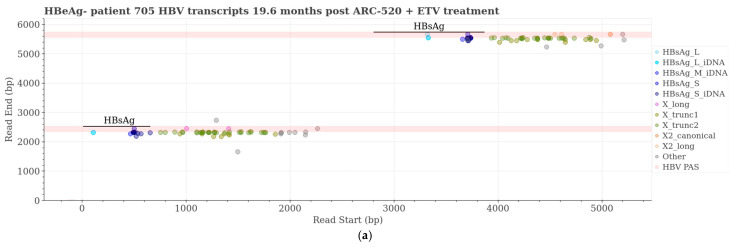

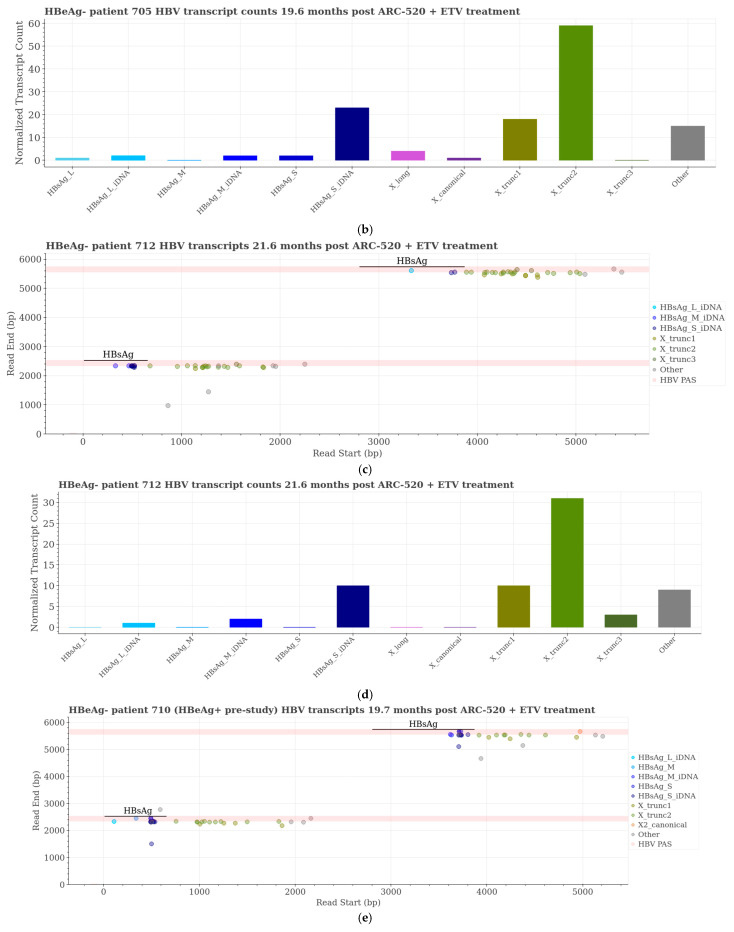

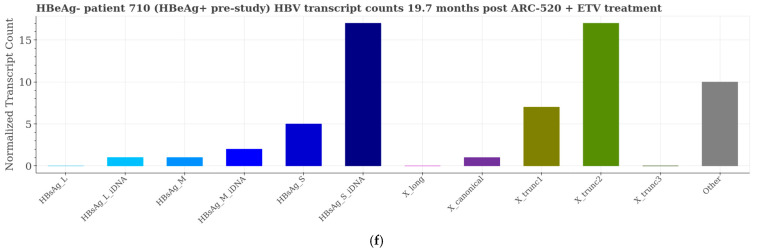
Patient HBV transcripts aligned by read start and read end positions on 2700–2200 concatemer of genotype B/C sequence AB073827 and identified transcript counts. Identified HBV transcripts aligned to the 2700–2200 concatemer are from HBeAg− patients 705 (**a**,**b**) and 712 (**c**,**d**), and previously HBeAg+ patient 710 (**e**,**f**). Pink bars, HBV PAS.

Unlike HBeAg− chimpanzees, X transcripts were much more abundant than S transcripts in the patients. The termination of most patient HBV transcripts prior to the HBV PAS is readily apparent from the alignment of the transcripts to the 2700–2200 HBV genome concatemer (Figure 8). In all the patient samples combined, a few S, a few long Xs, and two canonical X transcripts terminated at the HBV PAS. Most HBV transcripts terminated following the alternative PAS as previously described by Hilger et al. [21], approximately 122 nt prior to the HBV PAS, and included a short segment of host sequence after the PAS. Unlike the very small percentages of truncated X transcripts in chimpanzees, 60.6% and 62.1% of the HBV transcripts in HBeAg− patients 705 and 712, respectively, are truncated X (Table 4, Figure 8a–d). These transcripts are categorized as X_trunc2 in our algorithm and are expected to produce an HBx protein approximately 12 amino acids shorter than wild-type HBx. Each of the patients had some, though fewer, of the more truncated X transcripts (X_trunc1). Many of the patient transcripts that are identified as “Other” did not meet the specified transcript definitions; they had an X sequence that started after the X translation start site and were terminally truncated (see Supplementary Figure S4).

As previously reported, cohort 10 included three HBeAg+ patients and all had >8 log_10_ IU/mL serum HBV DNA prior to the start of the study (Supplementary Table S7). Patients 708 and 710 achieved undetectable serum HBV DNA 25.6 weeks after the SD (prior to MD) and 42.4 weeks after receiving MD ARC-520, respectively, and achieved seroclearance of HBeAg [31]. Patient 708 seroconverted for HBsAg 29.8 months after cessation of ARC-520. Patient 710, who had 80,918 IU/mL HBsAg at the baseline, had HBsAg reduced to 25.8 IU/mL at the last follow-up (Supplementary Table S7). Patients 708 and 710 achieved undetectable serum HBV RNA 11.9 and 50.3 weeks after the last MD, respectively, and HBcrAg decreased ≥ 5.1 log_10_ in both patients. Although patient 711 did not seroclear HBV products, she had significant decreases in all parameters at the last follow-up: −1.76 log_10_ IU/mL HBsAg, −3.13 log_10_ PEI U/mL HBeAg, −2.4 log_10_ kU/mL HBcrAg, −4.85 log_10_ IU/mL HBV DNA, and −1.4 log_10_ U/mL HBV RNA (Supplementary Table S7). Patient 711 had received six doses of ARC-520, whereas patients 708 and 710 had received eight doses. Of these three patients, only patient 710 had a biopsy and, thus, transcripts to analyze via Iso-seq (Figure 8e,f).

The reduction in viral parameters after MD ARC-520 treatment of patient 710 that was previously reported is shown for this patient in Supplementary Tables S7 and S8. Less than 5% of hepatocytes were HBsAg-positive upon histological evaluation. He had 3.4 copies/cell cccDNA and 5.3 copies/cell total HBV DNA at the follow-up, but we detected very few cccDNA-derived transcripts via Iso-seq analysis (Table 4). A total of 61 HBV transcripts were detected, similar to the two HBeAg− patients with detectable HBV transcripts and more than an order of magnitude less than in the highly viremic HBeAg+ chimpanzees. Only 7 (11.5%) of the 61 transcripts terminated with the HBV PAS and were, thus, classified as cccDNA-derived (Supplementary Table S9).

The 58, 17, and 40 HBV integration sites identified in patients 705, 710, and 712, respectively, are shown in Supplementary Table S10. Multiple transcripts from patients 705 and 710 (both males) were fused to microtubule-associated protein 4 (MAP4), glypican 6 (GPC6), GPC6-antisense RNA 1, and the VPS53 subunit of the Golgi-associated retrograde protein. Patient 705 also had multiple HBV transcripts fused to the albumin gene. Patient 712 (female) had fusions into different genes.

Consistent with none of the biopsied patient samples staining positive for HBcAg (core) and HBeAg being <LLOQ in each of these patients, no transcripts with the potential to encode HBeAg, core, or pgRNA were detected in any of the patients (Supplementary Table S9). Taken together, our results strongly suggest that cccDNA was silenced following MD ARC-520 treatment in conjunction with ETV.

## 4. Discussion

All sequenced transcripts from the liver biopsies of the HBeAg+ and HBeAg− chimpanzees and patients who had been treated with MD ARC-520 siRNAs in conjunction with ETV were aligned to a closely related HBV genome and characterized to identify the genes that they had the potential to encode. Multiple transcripts had the potential to encode each HBV protein in chronically HBV-infected chimpanzees: HBeAg, HBV core, HBsAg, HBV polymerase, and HBx. The sources of HBV proteins differed in the HBeAg+ compared to HBeAg− chimpanzees. The HBeAg+ chimpanzees had primarily cccDNA-derived transcripts with the potential to encode L-HBsAg, M-HBsAg, and S-HBsAg; HBeAg from precore mRNA and from splice products; core from pgRNA and from splice products; polymerase from pgRNA or POL (transcripts that started distal to DR1); and HBx primarily from long X transcripts. These chimpanzees had virtually no detectable canonical X in any samples, neither pre-treatment, on ETV alone, nor during the rebound post-treatment.

Interestingly, both the HBeAg+ and HBeAg− chimpanzees had transcripts that appeared to be from dslDNA dimers. Instead of integrating, dslDNA can circularize to form a cccDNA with deletion(s) or insertion(s) (“indels”) near DR1, a type of quasispecies [32]. Such quasispecies commonly have indels in the X ORF, which has its stop codon a few bases downstream of DR1. Our results suggest that dslDNA molecules can also circularize as dimers. The transcripts that contain two copies of the X gene (X_2X) were previously identified in hepatic cell lines that produced HBV particles and were thought to be the result of leaky termination [29,33]. To the best of our knowledge, this is the first time such X transcripts have been described in vivo. While it is possible that this 2X transcript results from leaky termination, this would not explain why the two copies of the X ORF are mostly not identical. RNA polymerase II, which transcribes the cccDNA and iDNA, has an error rate of 10^−5^ to 10^−6^ [34]. The Iso-seq method includes cDNA synthesis with an MMLV-based reverse transcriptase, but the point mutations and indels that we observe are unlikely to be introduced in this step because the error rate of this enzyme is approximately 10^−4^ [35]. X_2X transcripts with two different sequences in the X gene region would arise from the transcription of two dslDNA molecules that had ligated and circularized. The HBsAg_2X transcripts are also greater than one genome length but lack one or both HBV PAS regions. When the HBsAg_2X transcripts terminate at an HBV PAS, this suggests a circularized dimer. Other such transcripts terminate in the host sequence, indicating that the two copies of dslDNA had integrated. Transcripts from dslDNA dimers were not detected in any of the patients who received MD ARC-520 and whose replication was suppressed on ETV for a total of approximately three years prior to the biopsy. It is unknown if chronically infected humans have dslDNA dimers at an earlier stage of infection and prior to treatment with replication inhibitors.

Abundant HBsAg expression impacts many types of immune cells to impair the immune control of the virus [36,37,38,39]. In the HBeAg− chimpanzees, more than 90% of the transcripts encoded HBsAg, and these were primarily from iDNA. The HBeAg− chimpanzees produced HBsAg mostly from single integrated copies of dslDNA (68–86%), from cccDNA (6–18%), and from 2X dslDNA (5–8%). These chimpanzees expressed 0–1.7% truncated X and 0–1% cccDNA-derived X. Despite having some cccDNA-derived S transcripts (6–18% of total transcripts), the HBeAg− chimpanzees lacked not only precore, as expected, but also pgRNA. They had just a few terminally truncated transcripts that had the potential to encode core or pol. Suslov et al. reported the reduction in transcription from cccDNA as well as cccDNA loss with HBeAg seroconversion, consistent with our observations of the HBeAg− chimpanzees with low cccDNA and limited transcription of it [40].

In the chimpanzees, ETV treatment, siRNA + ETV treatment, and HBeAg-seroconversion each reduced cccDNA during 13 months of treatment, but in the absence of longer ETV treatment, the reduction was sustained in only one chimpanzee. Bowden et al. previously observed a decrease in cccDNA upon HBeAg loss in patients treated with ETV much longer, for 48 weeks [41]. The reduction in cccDNA was sustained off ETV in one chimpanzee that seroconverted for HBeAg but not in the other, highlighting the importance of longer-term treatment with ETV or other NA, especially as viral antigens rebound, and suggesting other factors, such as the immune system, are involved in maintaining the cccDNA reduction.

The treatment of high-viremia HBeAg+ patients with MD ARC-520 siRNA in conjunction with continued ETV after the siRNA has led to the transcriptional silencing of cccDNA, as others have demonstrated, occurs with NA treatment [42,43] or with siRNA treatment in a mouse model [13]. One patient (708) had achieved undetectable or <LLOQ HBV markers including HBsAg, seroconverted for HBsAg, and stopped ETV treatment [23]. The dramatic reduction in viral parameters began following the first dose of ARC-520. A second patient (710) also demonstrated a dramatic reduction in viral parameters, but in his case, these were especially notable following the MD ARC-520. The third patient (711) demonstrated deep reductions in all the viral parameters but not to the degree observed in the first two. It should be noted that the patients received from four to nine doses of ARC-520 treatment due to the termination of ARC-520 development, and patient 711 had only received six doses. One of these patients (710) consented to a biopsy and had sufficient liver RNA for Iso-seq characterization. Remarkably, he had 3.4 copies of cccDNA/cell with silenced cccDNA transcription. There was no earlier biopsy to determine whether cccDNA had decreased when he seroconverted for HBeAg. This cccDNA burden resembles that of the high-viremia HBeAg+ chimpanzees that did not experience cccDNA silencing and is two orders of magnitude more than the chimpanzee that achieved HBeAg− HBV infection with low serum HBV DNA and normalized ALT. A key difference between the chimpanzee study and the patient study was that patients remained on ETV for at least three years and were taken off ETV only after seroclearance of HBsAg and a year of consolidation therapy. This difference suggests that treatment with ETV to prevent new infection while viral antigens rebound post-siRNA treatment may be important for transcriptional silencing.

All three Heparc-2001 study patients that underwent a biopsy and had detectable HBV transcripts had similar HBeAg− transcript profiles with primarily HBV transcripts from iDNA, even though patient 710 originally had very high levels of all the viral parameters prior to starting the ARC-520 treatment. Iso-seq analysis from the biopsy at the last follow-up detected no pgRNA transcripts in any patient nor any precore. All the patients were or had become HBeAg−, so the lack of precore transcripts was unsurprising.

The most striking observation in the patients, and in contrast to the chimpanzees, was that these patients had high percentages of truncated X transcripts: 39.3% of the transcripts in the HBeAg+ patient that had transitioned to HBeAg− and 60.6% and 62.1% of the transcripts in the patients that were HBeAg− at the start of the study. The formerly HBeAg+ patient 710 had more S transcripts from iDNA (32.8%) than the two HBeAg− patients 705 and 712 who had 21.3% and 19.7%, respectively. Patient 710 was younger (23) than patients 705 (57) and 712 (40). The data suggest that the expression of truncated X increases with age while the proportion of HBsAg decreases, but there were too few patients in this study to determine if this truly is the case. As reviewed by Li et al., HBx transcripts truncated via termination at the alternative PAS likely retain the ability to bind DDB1 and destabilize Smc5/6; and the accumulation of this truncated HBx is likely to promote cell survival and clonal expansion [44]. The survival advantage of hepatocytes containing truncated HBx can explain the greater percentage of such transcripts in older patients. The presence of HBx in hepatocytes could make patients more vulnerable to reinfection with new cccDNA, and it is unknown how they could maintain transcriptionally silenced cccDNA in this situation. Patient 710 who had HBx transcripts from iDNA was able to achieve the substantial silencing of cccDNA. Perhaps the cells that express X from iDNA are not those that contain cccDNA. A known risk of having HBx expressed from integrated HBV is HCC [45], and seroclearance of HBsAg at a younger age reduces the HCC risk [46,47].

The silencing of cccDNA by using siRNA is an exciting prospect for the treatment of HBV patients, especially younger patients with high viremia who were previously not considered candidates for treatment. The presence of cccDNA remains a risk for reactivation, but the cccDNA can be lost over time if the cccDNA is silenced and not replenished. Age has been negatively correlated with HBsAg reduction following siRNA treatment [48]. Although T-cell responses do not appear to differ across the different stages of CHB [49], younger patients demonstrated broader T-cell function than older patients [50]. Our study demonstrates that most S and X transcripts in the HBeAg− patients are derived from iDNA. Treating patients at a younger age when HBV transcripts are primarily from cccDNA would limit the amount of integrated HBV that they will have when they are older.

The clinical development of ARC-520 was discontinued following a finding due to the EX1 excipient in an animal toxicology study; however, RNAi therapeutics without this excipient that are currently in clinical testing include ARO-HBV/JNJ-3989, ARB-729, RG6346 (RO7445482), and VIR-2218. Short-term treatment with JNJ-3989 (three monthly doses) resulted in reduced viral parameters in 38% of the patients a year later, suggesting that this HBV siRNA therapeutic also has the potential for transcriptional silencing [51]. Some of these patients also had a dramatic reduction in cccDNA-derived products, namely serum HBV RNA, HBcrAg, and HBeAg, but the number of patients with these dramatic reductions was limited. Longer continuous treatment with JNJ-3989 (48 weeks) did not result in higher numbers of patients with decreasing HBsAg post-siRNA, although there were some patients who responded with declining HBV parameters after the siRNA treatment stopped, while NA treatment continued for 24 weeks [52]. Clinical trial results with both ARC-520 and JNJ-3989 suggest that periodically repeated periods of short-term treatment with HBV siRNA and continuing NA treatment may lead to more functional cures in patients with cccDNA-driven HBV RNA and proteins. The excipient of ARC-520 may have played a role in the greater HBeAg+ patient response following ARC-520, compared to JNJ-3989, but the ARC-520 study was small. Achieving a functional cure in patients producing HBsAg and HBx from iDNA may require additional treatment beyond HBV siRNA plus NA.

## Figures and Tables

**Table 1:** Characteristics of chronically HBV-infected chimpanzees and treatment regimens. Animal identification, sex and age (years), HBeAg status, serum HBV DNA, and HBsAg are shown as the values on study day 1. ETV lead-in indicates the number of days that chimpanzees received daily oral entecavir (0.5 mg/daily for lamivudine-naïve animals; 1.0 mg/daily for lamivudine-experienced animals) prior to the first dose of ARC-520 (ARC-520 day 1). ARC-520 or other HBV siRNAs were given Q4W after the ETV lead-in period.

Animal ID	Sex/Age	HBeAg Status	Serum HBV DNA (Copies/mL)	Serum HBsAg (µg/mL)	ETV Lead-in (Days)	Doses siRNA (mg/kg × Number of Injections)
A2A004	M/12	Positive	8.5 log_10_	3188	85	3 mg/kg ARC-520 × 5; 4 mg/kg ARC-520 × 4
A3A006	M/10	Positive	8.3 log_10_	2104	141	4 mg/kg ARC-520 × 7
A4A014	F/9	Positive	7.7 log_10_	253	57	2 mg/kg ARC-520 × 2; 3 mg/kg ARC-520 × 4; 4 mg/kg ARC-520 × 4
89A008	M/24	Transitional	8.6 log_10_	245	141	4 mg/kg ARC-520 × 6; 4 mg/kg siHBV-75 × 1
88A010	M/25	Negative	3.0 log_10_	199	57	2 mg/kg ARC-520 × 2; 3 mg/kg ARC-520 × 4; 4 mg/kg ARC-520 × 1; 4 mg/kg siHBV-75 × 3; (2 mg/kg siHBV-75 + 2 mg/kg siHBV-74) × 1
95A010	F/18	Negative	3.5 log_10_	86	57	2 mg/kg ARC-520 × 2; 3 mg/kg ARC-520 × 4; 4 mg/kg ARC-520 × 1; 4 mg/kg siHBV-75 × 3; (2 mg/kg siHBV-75 + 2 mg/kg siHBV-74) × 1

**Table 2:** Chimpanzee sample time points, viral parameters, and Iso-seq transcript counts. HC (health check) was conducted 40 days prior to the study start date. ETV lead-in period varied from 57–141 days. The first day of ARC-520 administration was ARC-520 day 1. TND, target not detected.

							Iso-Seq Liver RNA Analysis		
Animal ID	Study Time Point	ARC-520 Time Point	On ETV at Time Point?	HBeAg (ng/mL)	Serum HBV DNA (copies/mL)	Serum HBsAg (µg/mL)	Total RNA Counts	HBV RNA Counts	Normalized HBV RNA Counts
88A010	d57	Day 1	Yes	TND	TND	195	288,459	565	385
95A010	d57	Day 1	Yes	TND	TND	78	250,796	107	85
89A008	HC (d−40)	Day −180	No	3797	8.6 log_10_	299	212,048	677	631
89A008	d141	Day 1	Yes	1.2	4.5 log_10_	151	253,594	372	289
89A008	d379	Day 239	No	TND	6.3 log_10_	134	248,962	294	232
A2A004	HC (d−40)	Day −124	No	10,348	8.2 log_10_	3684	243,360	2824	2281
A2A004	d85	Day 1	Yes	7538	5.0 log_10_	2866	196,578	2557	2557
A2A004	d323	Day 239	No	810	2.7 log_10_	13	219,019	72	68
A2A004	d351	Day 267	No	2132	8.3 log_10_	86	211,714	448	420
A3A006	HC (d−40)	Day −180	No	7508	8.3 log_10_	1951	218,337	2136	1930
A3A006	d141	Day 1	Yes	4839	4.7 log_10_	1640	309,355	2956	1881
A3A006	d351	Day 211	No	2591	8.9 log_10_	318	223,312	1297	1143
A4A014	HC (d−40)	Day −96	No	4.7	7.6 log_10_	242	251,414	628	494

**Table 3:** Transcript name, description, counts, and percentages are shown for the combined HBeAg+ compared to HBeAg− chimpanzee HBV transcriptomes. The same type of transcript aligned in two locations on the 2700–2100 concatemer template are combined in this table, e.g., HBsAg_L1 and HBsAg_L2 are counted together as HBsAg_L.

		HBeAg+		HBeAg−	
Transcript Name	Description of the Potential Protein Product	Count	% Total	Count	% Total
HBeAg_wt	Wild-type precore	2166	18.57	0	0
HBeAg_ORF	HBeAg ORF encoded but terminates prior to PAS	11	0.09	0	0
HBeAg_sp6	Entire HBeAg ORF followed by 2471/489 splice	17	0.15	0	0
HBeAg_sp11	Entire HBeAg ORF followed by 2471/282 splice	12	0.1	0	0
HBeAg_sp1_Cys−	HBeAg ORF lacking terminal cysteine	83	0.71	0	0
HBeAg-S_sp5	Fusion of HBeAg and HBsAg due to 2087/489 splice	12	0.1	0	0
HBeAg-S_sp9	Fusion of HBeAg and HBsAg due to 2447/282 splice	80	0.69	0	0
POL	HBV polymerase ORF encoded, termination after ORF and up to HBV PAS	83	0.71	2	0.29
Core_wt	pgRNA encoding core and polymerase	1690	14.49	0	0
Core_ORF	Core ORF encoded but terminates prior to PAS	10	0.09	3	0.43
Core_sp6	Entire Core ORF followed by 2471/489 splice	18	0.15	0	0
Core_sp11	Entire Core ORF followed by 2471/282 splice	9	0.08	0	0
Core-sp1_Cys−	Core ORF lacking a terminal cysteine	89	0.76	0	0
Core-S_sp5	Fusion of core and HBsAg due to 2087/489 splice	6	0.05	0	0
Core-S_sp9	Fusion of core and HBsAg due to 2447/282 splice	106	0.91	0	0
S-Core	Fusion of HBsAg and core due to 458 splice in the 2X genome	4	0.03	0	0
HBsAg_L	L-HBsAg with an HBV PAS terminus	625	5.36	6	0.86
HBsAg_L_iDNA	L-HBsAg from iDNA	26	0.22	15	2.14
HBsAg_M	M-HBsAg with an HBV PAS terminus	495	4.24	2	0.29
HBsAg_M_iDNA	M-HBsAg from iDNA	14	0.12	4	0.57
HBsAg_S	S-HBsAg with an HBV PAS terminus	4884	41.86	106	15.12
HBsAg_S_iDNA	S-HBsAg from iDNA	460	3.94	471	67.19
HBsAg_2X_1	L-, M-, or S-HBsAg ORF followed by deletion, terminating at HBV PAS	0	0	0	0
HBsAg_2X_2	L-, M-, or S-HBsAg ORF followed by a large deletion, terminating 294–1194 bases after HBV PAS	9	0.08	15	2.14
HBsAg_2X_3	L-, M-, or S-HBsAg ORF followed by deletion, terminating between the second S gene and HBV PAS on the 2X genome	27	0.23	27	3.85
HBsAg_2X_4	L-, M-, or S-HBsAg ORF followed by deletion, terminating after the second HBV PAS on 2X genome	13	0.11	7	1
X_canonical	Canonical X gene product	1	0.01	0	0
X_long	Long X transcript with a start site after that of S-HBsAg, with an HBV PAS terminus	342	2.93	3	0.43
X_trunc1	X transcript that is truncated at the 3′ terminus just after DR2	3	0.03	1	0.14
X_trunc2	X transcript with 66 or less amino acid residues truncated at the 3′ terminus	10	0.09	8	1.14
X_trunc3	X transcript with complete ORF but terminates prior to HBV PAS	2	0.02	1	0.14
X_2X	Long X transcript that starts after the S stop codon and terminates after passing the HBV PAS twice	31	0.27	0	0
Other	Transcripts that do not meet any of the predefined criteria	329	2.82	30	4.28
Total		11,667	100.01	701	100.01

**Table 4:** Major HBV S and X transcript counts and percentages in each patient sample. HBV transcripts that had the potential to encode the complete ORF of HBsAg and HBx were counted in each patient RNA sample. Shown are the normalized transcript counts and the percentage of that transcript within each sample. The total number of S transcripts is a combination of those that include the HBV PAS and are presumed to be derived from cccDNA (HBsAg_L, HBsAg_M, HBsAg_S) and those that terminate after the S ORF but prior to the HBV PAS that are presumed to be from iDNA (HBsAg_L_iDNA, HBsAg_M_iDNA and HBsAg_S_iDNA). Full-length HBx transcripts are defined as those with complete HBx ORF (X_canonical, X_long, and X_trunc3). Truncated X are X_trunc1 and X_trunc2.

Patient no. (HBeAg Status at the Baseline)	Total Normalized Transcript Counts	HBsAg Transcripts						HBV X Transcripts			
		Total HBsAg		HBsAg_iDNA		HBsAg_cccDNA		Total X Full Length		Truncated X	
		Counts	%	Counts	%	Counts	%	Counts	%	Counts	%
705 (neg)	127	30	23.6%	27	21.3%	3	2.4%	5	3.9%	69	60.6%
710 (pos)	61	26	42.6%	20	32.8%	6	9.8%	1	1.6%	20	39.3%
712 (neg)	66	13	19.7%	13	19.7%	0	0.0%	3	4.5%	39	62.1%

## Data Availability

The chimpanzee Iso-seq transcriptome data have been deposited in NCBI’s Gene Expression Omnibus and are accessible through GEO Series accession number GSE279971 (https://www.ncbi.nlm.nih.gov/geo/query/acc.cgi?acc=GSE279971 (accessed on 17 December 2024)). Patient HBV transcriptomes are available in the Supplementary Materials, but the whole transcriptomes will not be available due to privacy concerns.

## Supplementary Materials

The following supporting information can be downloaded at https://www.mdpi.com/article/10.3390/v16121943/s1, Supplementary Methods; Supplementary Results; Table S1: Quantification of chimpanzee liver HBV DNA with and without DNase treatment; Table S2: HBV transcript definitions for chimpanzee and human HBV; Table S3 (Excel file): HBV transcript counts and percentages for all chimpanzee samples combined and each individual chimpanzee sample; Table S4 (Excel file): Sequences and transcript categorization of all chimpanzee sample transcripts; Table S5: Major HBV X and S transcript counts and percentages in each chimpanzee sample; Table S6: Precore/core transcript counts and percentages in each chimpanzee sample; Table S7: Demographics, treatment details, and viral parameters for the eight patients in cohort 10; Table S8: Intrahepatic virologic profile, immunohistochemical staining and total Iso-seq transcript counts from liver biopsies at last follow-up for five patients; Table S9 (Excel file): Sequences and transcript categorization of all human patient HBV transcripts; Table S10 (Excel file): HBV chromosome integration sites in patient liver transcripts; Figure S1: Alignment of chimpanzee A4A014_HC transcripts to HBV genome concatemers 1600–2100 and 2700–2100; Figure S2: Chimpanzee HBV transcripts aligned by read start and read end positions on 2700–2100 concatemer and normalized transcript counts; Figure S3: Sequences of first (A) and second (B) HBx open reading frames in the X_2X transcript of chimpanzee A3A006, study day 141 after the ETV lead-in; Figure S4: Alignment of Iso-seq HBV transcripts from Heparc-2001 study patients. References in Supplementary Materials: [17,53,54,55,56].
