# Supplementary material for: Characterization of Hepatitis B Virus Transcripts in Chronically HBV-Infected Chimpanzees and Patients Treated with ARC-520 siRNA Demonstrates Transcriptional Silencing of cccDNA
Source: Viruses. 2024 Dec 19;16(12):1943. doi: 10.3390/v16121943 (PMC11680220; doi:10.3390/v16121943)
Supplement: Supplementary file 1 [file viruses-16-01943-s001.zip › 20241217_Supplementary Materials_revised.pdf]

Supplementary Materials

Characterization of Hepatitis B virus transcripts in chronically HBV-infected chimpanzees and patients treated with ARC-520 HBV siRNA demonstrates transcriptional silencing of cccDNA

Christine I. Wooddell, Dean Sanders, Zhao Xu, Lung-Yi Mak, Thomas Schluep, Wai-Kay Seto, Bruce D. Given and Man-Fung Yuen

This PDF file includes supplementary methods, tables and figures.

Supplementary Materials and Methods

Chimpanzee HBV reference sequences and genome assembly

The consensus sequences for the six chimpanzee HBV genomes in this study were determined by next generation sequencing of both their genomic HBV DNA and their HBV RNA in liver samples after ETV lead-in but prior to ARC-520 treatment ("ARC-520 Day 1") as previously described [1]. The HBV sequences of the six chimpanzees differed by only 2–4 bases from that of chimpanzee ID 88A010, and all HBV transcripts from each animal in this study were aligned to the sequence of chimpanzee 88A010.

HBV from these chimpanzees and the human reference GenBank accession number V01460 have a circular genome of 3,182 base-pairs (bp) that is transcribed more than one genome length to produce the precore and pgRNA transcripts generally named "3.5 kb". From precore transcription start site at HBV position 1781 to the end of the HBV PAS at 1919, the sequence comprises positions 1781–3182 + 1–1919 (3321 bases) plus variable sequence beyond the PAS and a polyadenylated tail.

Chimpanzee liver HBV DNA quantitation and analysis of DNA form

As previously reported [1], snap frozen liver biopsies were ground in PBS using a sterile, RNAse-free disposable pellet pestle and extracted for DNA using the QIAamp DNA mini kit (Qiagen, Cat. No. 51304, Germantown, MD, USA) following the manufacturer's protocol. DNA concentration was determined using a Nanodrop ND-1000 and 250 ng of liver DNA was used per quantitative PCR TaqMan assay, in duplicate, using the core forward primer CGAGGCAGGTCCCCTAGAAG and core reverse primer TGCGACGCGGYGATTG (300 nM final concentration of each) and core probe (FAM/TAMRA dual-labeled) AGAACTCCCTCGCCTCGCAGACG (200 nM final) to a conserved region in the HBV core gene, and the TaqMan Fast Advanced DNA kit (Applied Biosystems, Cat. No. 4444557, Waltham, MA, USA). Y represents either pyrimidine to account for HBV strain differences. All TaqMan assays were performed with an Applied Biosystem (ABI) 7500 Sequence Analyzer. All primers and probes were from Integrated DNA Technologies (Coralville, IA, USA).

The plus DNase samples were processed using the Plasmid Safe, ATP dependent DNase kit (Epicentre/Illumina, Cat. No. E3105K, Madison, WI, USA) and 250 ng liver DNA in a 5 µL volume

was mixed with 15 µL of the DNase mix, incubated for 30 min at 37°C, then heat-inactivated for 30 min at 70°C. The entire 20 µL volume was utilized in a 50 µL total volume TaqMan assay.

Measurement of intrahepatic HBV DNA, cccDNA, and pgRNA in Heparc2001 patients

Liver needle biopsy tissue was homogenized in the presence of RNeasy lysis buffer. The lysate was split into 2 equal parts, one for total DNA and one for RNA extraction. Total liver RNA was extracted by the RNeasy Extraction Kit (Qiagen, Hilden, Germany). Total liver DNA extraction was performed with the QiaAmp Mini Kit (Qiagen) after pH adjustment of the lysate with 1M Tris/HCl pH 8. Real-time quantification of intrahepatic total HBV DNA and cccDNA were performed as previously described [2, 3], with a slight modification that Exonuclease I and Exonuclease III (both New England Biolabs, Ipswich, MA, USA) was used in place of Plasmid-Safe DNase for the enrichment of cccDNA. Real-time quantification of the pgRNA was performed as previously described [4].

Supplementary Results

Neoantigens

This analysis identifies transcripts by starting and ending positions, but it must be noted that many transcripts have an abundance of single base insertions, deletions and point mutations that repeatedly shift the reading frame and alter the protein sequence, resulting in an abundance of neoantigens. These can be visualized in Figures 3 and 4 and Supplementary Figure S1. HBV's high replication rate and error-prone reverse transcription lead to a heterogeneous viral population of closely related but not identical sequences, thus named quasispecies [5]. Quasispecies have limited ability to replicate, due to the indels, but they are transcribed and are a source of neoantigens. The overlapping reading frames of HBV facilitate fusions of HBeAg+pol, core+pol, HBsAg+pol and pol+HBx. The sp5 and sp9 splicing of the precore or pgRNA transcripts can produce fusion proteins of HBeAg+HBsAg or core+HBsAg, respectively. With frame shifts, these can become HBeAg+pol+HBsAg in alternating segments of the polypeptide.

Supplementary Table S1. Quantification of chimpanzee liver HBV DNA with and without DNase treatment. The Plasmid Safe, ATP-dependent DNase treatment (+DNase) as described in the Materials and Methods is a measurement of cccDNA.

Chimpanzee ID/time point	No DNase		+DNase		Fold-change +DNase	Dosing notes	HBeAg (ng/ml)
	Log_{10} copies/ µg DNA	Log change from Baseline	Log_{10} copies/ µg DNA	Log change from Baseline			
A2A004/d −8	6.86	BL	5.78	BL	−12.1	Pre-Study	11,360
A2A004/d85	5.68	−1.2	5.47	−0.3	−1.6	85 days ETV/pre-ARC-520 #1	9810
A2A004/d120	5.68	−1.2	5.31	−0.5	−2.3	120 days ETV/ 2 weeks post-ARC-520 #2	1359
A2A004/d295	5.20	−1.7	5.10	−0.7	−1.3	295 days ETV/ 2 weeks post-ARC-520 #8	259
A2A004/d323	5.02	−1.8	4.77	−1.0	−1.8	5 days off ETV/2 weeks post-ARC-520 #9	984
A2A004/d351	6.68	−0.2	5.45	−0.3	−17.0	33 days off ETV/6 weeks pos-ARC-520 #9	3583
A2A004/d379	7.21	0.3	6.14	0.4	−11.6	61 days off ETV/10 weeks post-ARC-520 #9	21,793
A3A006/d −8	6.78	BL	5.42	BL	−23.1	Pre-Study	8874
A3A006/d141	5.66	−1.1	5.27	−0.1	−2.4	141 days ETV/pre-ARC-520 #1	5548
A3A006/d176	5.57	−1.2	5.29	−0.1	−1.9	176 days ETV/2 weeks post-ARC-520 #2	<LLOD
A3A006/d295	5.29	−1.5	5.04	−0.4	−1.8	295 days ETV/2 weeks post-ARC-520 #6	84
A3A006/d323	5.65	−1.1	5.05	−0.4	−4.0	5 days off ETV/2 weeks post-ARC-520 #7	322
A3A006/d351	7.24	0.5	6.04	0.6	−16.0	33 days off ETV/6 weeks post-ARC-520 #7	4600
A3A006/d379	7.34	0.6	6.17	0.7	−14.8	61 days off ETV/10 weeks post-ARC-520 #7	15,098
A4A014/d −8	5.93	BL	4.76	BL	−14.8	Pre-Study	4.3
A4A014/d57	4.71	−1.2	4.54	−0.2	−1.5	57 days ETV/pre-ARC-520 #1	6
A4A014/d92	4.56	−1.4	4.49	−0.3	−1.2	92 days ETV/2 weeks post-ARC-520 #2	0.8
A4A014/d176	4.37	−1.6	4.25	−0.5	−1.3	176 days ETV/2 weeks post -ARC-520 #5	<LLOD
A4A014/d265	3.78	−2.1	3.85	−0.9	1.2	265 days ETV/2 weeks post-ARC-520 #8	<LLOD (NEG since d183, Ab+)
A4A014/d323	3.62	−2.3	3.45	−1.3	−1.5	5 days off ETV/2 weeks post-ARC-520 #10	<LLOD
A4A014/d351	3.58	−2.3	3.21	−1.5	−2.3	33 days off ETV/6 weeks post-ARC-520 #10	<LLOD
A4A014/d379	3.88	−2.0	3.70	−1.1	−1.5	61 days off ETV/10 weeks post-ARC-520 #10	<LLOD
88A010/d −8	4.45	BL	2.21	BL	−175.4	Pre-Study	NEG/Ab+
88A010/d57	4.11	−0.3	2.15	−0.1	−92.3	57 days ETV/pre-ARC-520 #1	NEG/Ab+
88A010/d92	4.20	−0.2	2.34	0.1	−72.3	92 days ETV/2 weeks post-ARC-520 #2	NEG/Ab+
88A010/d265	3.77	−0.7	2.03	−0.2	−55.0	265 days ETV/2 weeks post-siHBV-75 #1	NEG/Ab+
88A010/d323	4.41	0.0	2.47	0.3	−87.6	323 days ETV/2 weeks post-siHBV-75 #3	NEG/Ab+
88A010/d351	4.38	−0.1	2.55	0.3	−66.8	351 days ETV/2 weeks post-siHBV-75/siHBV-74	NEG/Ab+
88A010/d379	4.24	−0.2	2.54	0.3	−49.6	28 days off ETV/6 weeks post-siHBV-75/siHBV-74	NEG/Ab+
89A008/d −8	7.10	BL	5.32	BL	−60.7	Pre-Study	4382.4
89A008/d141	4.65	−2.5	4.04	−1.3	−4.0	141 days ETV/pre-ARC-520 #1	1.2
89A008/d176	4.57	−2.5	3.91	−1.4	−4.6	176 days ETV/2 weeks post -ARC-520 #2	<LLOD (NEG since d148, Ab+)
89A008/d295	4.18	−2.9	3.39	−1.9	−6.2	295 days ETV/2 weeks post-ARC-520 #6	<LLOD
89A008/d323	4.56	−2.5	3.28	−2.0	−19.0	323 days ETV/2 weeks post-siHBV-75 #1	<LLOD
89A008/d351	4.77	−2.3	3.70	−1.6	−11.7	28 days off ETV/6 weeks post-siHBV-75 #1	<LLOD
89A008/d379	4.72	−2.4	3.79	−1.5	−8.4	56 days off ETV/10 weeks post-siHBV-75 #1	<LLOD

95A010/d −8	3.59	BL	2.87	BL	−5.3	Pre-Study	NEG/Ab+
95A010/d57	3.57	0.0	2.89	0.0	−4.7	57 days ETV/pre-ARC-520 #1	NEG/Ab+
95A010/d92	3.80	0.2	3.13	0.3	−4.7	92 days ETV/2 weeks post-ARC-520 #2	NEG/Ab+
95A010/d265	3.14	−0.4	2.29	−0.6	−7.1	265 days ETV/2 weeks post-siHBV-75 #1	NEG/Ab+
95A010/d323	3.23	−0.4	3.35	0.5	1.3	323 days ETV/2 weeks post-siHBV-75 #3	NEG/Ab+
95A010/d351	3.27	−0.3	3.17	0.3	1.2	351 days ETV/2 weeks post-siHBV-75/siHBV-74	NEG/Ab+
95A010/d379	3.61	0.0	3.28	0.4	−2.1	28 days off ETV/6 weeks post-siHBV-75/siHBV-74	NEG/Ab+

LLOD, lower limit of detection for HBeAg was <0.4 ng/ml; NEG, HBeAg-negative; Ab+, positive for anti-HBeAg antibodies

Supplementary Table S2. HBV transcript definitions for chimpanzee and human HBV. Start site locations, open reading frame (ORF), splice donor and splice acceptor sites are indicated for the 1X chimpanzee or human (GenBank accession # AB073827) genome and in the ~2x genomes (2700–2100 or 2700–2200).

**HBV transcripts**	**Chimpanzee genome 1X**	**Chimpanzee genome 2700–2100**	**Human genome 1X**	**Human genome 2700–2200**
HBsAg-L start	2700–2835	NA	2700–2833	NA
HBsAg-L ORF	2850–834	NA	2848–832	NA
HBsAg-L1 start	NA	1–136	NA	1–134
HBsAg-L1 ORF	NA	151–1317	NA	149–1348
HBsAg-L2 start	NA	3183–3318	NA	2804–3349
HBsAg-L2 ORF	NA	3333–4499	NA	3364–4563
HBsAg-M start	2836–3154	NA	2834–3185	NA
HBsAg-M ORF	3174–834	NA	3205–832	NA
HBsAg-M1 start	NA	137–455	NA	135–486
HBsAg-M1 ORF	NA	475–1317	NA	506–1348
HBsAg-M2 start	NA	3319–3637	NA	3350–3701
HBsAg-M2 ORF	NA	3657–4499	NA	3721–4563
HBsAg-S start	3155–142	NA	3186–140	NA
HBsAg-S	157–834	NA	155–832	NA
HBsAg-S1 start	NA	456–625	NA	487–656
HBsAg-S1	NA	640–1317	NA	671–1348
HBsAg-S2 start	NA	3628–3807	NA	3702–3871
HBsAg-S2	NA	3822–4499	NA	3886–4563
HBeAg start	1397-1796	1880–2279	1395–1794	1911–2310
HBeAg	1816–2451	2299–2934	1814–2449	2330–2965
pgRNA start	1797–1822	2280–2305	1795–1820	2311–2336
pgRNA end	1923–2100	5588–5765	1921–2200	5652–5931
Core start	1797–1883	2280–2366	1795–1881	2311–2397
Core ORF	1903–2451	2386–2934	1901–2449	2417–2965
Polymerase start	1889–2294	2372–2777	1882–2287	2398–2803
Polymerase	2309–1622	2792–5287	2307–1623	2823–5354
X canonical start	1240–1361	NA	1238–1359	NA
X-long start	143–1239	NA	141–1237	NA
X ORF	1376–1837	NA	1374–1835	NA
X1 canonical start	NA	1723–1844	NA	1754–1875
X1-long start	NA	626–1722	NA	657–1753
X1 ORF	NA	1859–2320	NA	1890–2351
X2 canonical start	NA	4905–5026	NA	4969–5090
X2-long start	NA	3808–4904	NA	3872–49
X2 ORF	NA	5041–5502	NA	5105–5566
**Splice donor and acceptor sites**	**Chimpanzee genome 1X**	**Chimpanzee genome 2700–2100**	**Human genome 1X**	**Human genome 2700–2200**
sp1 donor 2447	2449	2932	2447	2963
sp1 acceptor 489	491	4156	489	4220
sp1 acceptor 489 in concatemer genome	491	974	489	1005
sp3 donor 2067	2069	2552	2067	2583
sp3 acceptor 489	491	4156	489	4220
sp5 donor 2087	2089	2572	2087	2603
sp5 acceptor 489	491	4156	489	4220

sp6 donor 2471	2473	2956	2471	2987
sp6 acceptor 489	491	4156	489	4220
sp9 donor 2447	2449	2932	2447	2963
sp9 acceptor 282	284	3949	282	4013
sp11 donor 2471	2473	2956	2471	2987
sp11 acceptor 282	284	3949	282	4013

Supplementary Excel Table S3. HBV transcript counts and percentages for all chimpanzee samples combined and each individual chimpanzee sample.

Supplementary Excel Table S4. Sequences and transcript categorization of all chimpanzee sample transcripts.

Supplementary Table S5. Major HBV X and S transcript counts and percentages in each chimpanzee sample. HBV transcripts that had the potential to encode the complete ORF of HBx and HBsAg were counted in each RNA sample, identified by chimpanzee and biopsy time point. Shown are the normalized transcript counts and the percentage of that transcript within each sample. Full-length X transcripts are defined as those with complete HBx ORF (X_canonical, X_long, X_2X and X_trunc3). Truncated X are X_trunc1 and X_trunc2. The total number of S transcripts is a combination of those that include the HBV PAS (HBsAg_L, HBsAg_M, HBsAg_S), those that terminate after the S ORF but prior to the HBV PAS that are presumed to be from iDNA (HBsAg_L_iDNA, HBsAg_M_iDNA and HBsAg_S_iDNA), and the HBsAg transcripts that are greater than one genome length (HBsAg_2X).

Chimpanzee Sample (HBeAg +/−)	X transcripts				HBsAg transcripts							
	Full length X		Truncated X		Total HBsAg		HBsAg_iDNA		HBsAg_cccDNA		HBsAg_2X	
	Counts	%	Counts	%	Counts	%	Counts	%	Counts	%	Counts	%
88A010_d57 (−)	4	1.04%	5	1.30%	355	92.21%	260	67.53%	68	17.66%	27	7.01%
95A010_d57 (−)	0	0.00%	0	0.00%	82	96.47%	73	85.88%	5	5.88%	4	4.71%
89A008_HC (+)	21	3.33%	6	0.95%	498	78.92%	196	31.06%	288	45.64%	14	2.22%
89A008_d141 (+/−)	4	1.38%	3	1.04%	267	92.39%	185	64.01%	65	22.49%	17	5.88%
89A008_d379 (−)	1	0.43%	4	1.72%	216	93.10%	157	67.67%	41	17.67%	18	7.76%
A2A004_HC (+)	82	3.59%	1	0.04%	1437	63.00%	16	0.70%	1420	62.25%	1	0.04%
A2A004_d85 (+)	88	3.44%	0	0.00%	1662	65.00%	18	0.70%	1640	64.14%	4	0.16%
A2A004_d323 (+)	2	2.94%	0	0.00%	28	41.18%	2	2.94%	26	38.24%	0	0.00%
A2A004_d351 (+)	15	3.57%	0	0.00%	180	42.86%	8	1.90%	172	40.95%	0	0.00%
A3A006_HC (+)	67	3.47%	1	0.05%	929	48.13%	18	0.93%	905	46.89%	6	0.31%
A3A006_d141 (+)	57	3.03%	0	0.00%	800	42.53%	14	0.74%	783	41.63%	3	0.16%
A3A006_d351 (+)	49	4.29%	0	0.00%	466	40.77%	16	1.40%	449	39.28%	1	0.09%
A4A014_HC (+)	18	3.64%	2	0.40%	286	57.89%	27	5.47%	256	51.82%	3	0.61%

Supplementary Table S6. Precore/core transcript counts and percentages in each chimpanzee sample. HBV transcripts with the potential to encode the complete ORF of HBeAg or core and those lacking the terminal cysteine due to sp1 splicing were counted in each RNA sample, identified by chimpanzee, biopsy time point and HBeAg status at that time point. Shown are the normalized transcript counts and the percentage of that transcript within each sample. Full-length HBeAg transcripts include the wild-type precore (HBeAg_wt), transcripts with complete ORF but early termination (HBeAg_ORF), and splice products (HBeAg_sp6 and HBeAg_sp11). Similarly, the total core includes pgRNA (core_wt), core_ORF, and core splice products (core_sp6 and core_sp11). HBeAg_Cys− and core_Cys− are precore and core products of sp1 splicing.

		Total HBeAg				Total Core			
		HBeAg		HBeAg_Cys−		Total Full-length Core		Core_Cys−	
Chimpanzee sample	HBeAg status	Counts	%	Counts	%	Counts	%	Counts	%
88A010_d57	−	0	0.00%	0	0.00%	1	0.26%	0	0.00%
95A010_d57	−	0	0.00%	0	0.00%	0	0.00%	0	0.00%
89A008_HC	+	17	2.69%	0	0.00%	46	7.29%	2	0.32%
89A008_d141	+/−	1	0.35%	0	0.00%	2	0.69%	0	0.00%
89A008_d379	−	0	0.00%	0	0.00%	2	0.86%	0	0.00%
A2A004_HC	+	391	17.14%	17	0.75%	272	11.92%	20	0.88%
A2A004_d85	+	394	15.41%	19	0.74%	250	9.78%	19	0.74%
A2A004_d323	+	9	13.24%	0	0.00%	12	17.65%	1	1.47%
A2A004_d351	+	76	18.10%	5	1.19%	108	25.71%	2	0.48%
A3A006_HC	+	500	25.91%	14	0.73%	300	15.54%	5	0.26%
A3A006_d141	+	481	25.57%	17	0.90%	390	20.73%	22	1.17%
A3A006_d351	+	255	22.31%	5	0.44%	271	23.71%	10	0.87%
A4A014_HC	+	82	16.60%	6	1.21%	76	15.38%	8	1.62%

Supplementary Table S7. Demographics, treatment details and viral parameters for the eight patients in cohort 10. Reproduced from [6] with permission from BMJ Publishing Group Ltd.

Patient no (# of MD)	Sex/ age	HBeAg / ALT (U/L)	Months from last MD to LFU	Time points	Virologic Parameters				
					HBsAg (IU/mL)	HBeAg (PEI U/mL)	HBcrAg (kU/mL)	HBV DNA (log IU/mL)	HBV RNA (log U/mL)
708 (8)	F/31	pos/ 31	29.1	Baseline	34388	1050	378470	8.54	6.39
				Start of MD	192	0.6	375.2	<LLOQ	2.56
				Nadir	<LLOQ	<LLOQ	1.4	TND	TND
				LFU	<LLOQ*	<LLOQ	1.4	TND	TND
710 (8)	M/23	pos/ 16	31.1	Baseline	80918	2970	883840	8.85	9.96
				Start of MD	13742	1040	648730	2.65	6.39
				Nadir	11	<LLOQ	5.9	TND	TND
				LFU	25.8	<LLOQ	7	<LLOQ	<LLOQ
711 (6)	F/36	pos/ 29	30.3	Baseline	64167	8934	579160	8.69	6.77
				Start of MD	17587	102	144490	3.57	6.61
				Nadir	821	13.9	6260.9	1.42	4.82
				LFU	1113	6.57	2295	1.79	4.83
701 (8)	M/57	neg/ 15	30.4	Baseline	6.87	<LLOQ	0.3	3.41	<LLOQ
				Start of MD	5.59	<LLOQ	1.3	<LLOQ	1.81
				Nadir	1.27	<LLOQ	0.2	TND	TND
				LFU	1.92	<LLOQ	0.6	TND	TND
705 (8)	M/57	neg/ 21	29.0	Baseline	4085	<LLOQ	19	5.16	2.51
				Start of MD	3522	<LLOQ	26.5	<LLOQ	1.83
				Nadir	451	<LLOQ	8.9	TND	2.06
				LFU	2677	<LLOQ	9	TND	2.27
706 (4)	M/37	neg/ 20	30.2	Baseline	1256	<LLOQ	0.6	4.26	1.92
				Start of MD	1035	<LLOQ	1.1	<LLOQ	1.81
				Nadir	136	<LLOQ	TND	TND	TND
				LFU	425	<LLOQ	TND	<LLOQ	<LLOQ
709 (5)**	M/45	neg/ 25	30.3	Baseline	10	<LLOQ	0.3	3.76	2.05
				Start of MD	2.1	<LLOQ	0.7	<LLOQ	<LLOQ
				Nadir	<LLOQ	<LLOQ	TND	TND	TND
				LFU	<LLOQ ^	<LLOQ	TND	<LLOQ	TND
712 (9)	F/40	neg/ 17	28.9	Baseline	2016	<LLOQ	2.1	4.37	2.25
				Start of MD	1896	<LLOQ	1.7	<LLOQ	<LLOQ
				Nadir	518	<LLOQ	0.4	TND	TND
				LFU	1018	<LLOQ	0.6	TND	1.82

Baseline is defined as the start of SD.
MD: multiple dose; LFU: last follow-up; LLOQ: lower limit of quantification (HBsAg < 0.05 IU/mL; HBeAg < 0.01 PEI U/mL; HBcrAg < 1 kU/mL; HBV DNA < 10 IU/ml; HBV RNA < 1.65 log U/mL); TND: Target not detected
Anti-HBs level: *25.12 IU/L; ^152.5 IU/L; **Entecavir was stopped 12 months after HBsAg seroclearance

Supplementary Table S8. Intrahepatic virologic profile, immunohistochemical staining and total Iso-seq transcript counts from liver biopsies at last follow up for five patients. Partially reproduced from [6] with permission from BMJ Publishing Group Ltd.

Patient no. (HBeAg status at baseline)	Time from last MD to liver biopsy (months)	Serum virologic parameters*		Intrahepatic virologic levels (copies/cell)			% of positive hepatocytes		Total Iso-seq counts
		HBsAg (IU/mL)	HBV DNA	Total HBV DNA	cccDNA	pgRNA	HBsAg	HBcAg	
701 (neg)	19.6	1.5	<LLOQ	0.985	0.382	0.082	10	0	274,466
705 (neg)	19.6	2305	TND	4.381	1.025	0.721	40	0	342,581
709 (neg)	22.9	<0.05	TND	0.947	0.263	0.096	0	0	308,447
710 (pos)	19.7	11.5	<LLOQ	5.326	3.425	0.090	<5	0	391,596
712 (neg)	21.6	778.6	TND	1.837	1.085	0.119	40	0	285,310

MD: multiple dose; LLOQ: lower limit of quantification (HBV DNA < 10 IU/mL); TND: Target not detectable
*Serum virologic parameters measured within 3 weeks of the liver biopsy
Immunohistochemical staining positivity stated to the nearest 10% unless otherwise specified

Supplementary Excel Table S9. Sequences and transcript categorization of all human patient HBV transcripts.

Supplementary Excel Table S10. HBV-chromosome integration sites in patient liver transcripts.

Supplementary Figure S1. Alignment of chimpanzee A4A014_HC transcripts to HBV genome concatemers 1600–2100 and 2700–2100. HBV transcripts from HBeAg-positive chimpanzee A4A014 liver biopsy collected at health check prior to any treatment were aligned to the HBV genome concatemer from position 1600–3182/1–2100 (A) or 2700–3182/1–3182/1–2100 (B). Open reading frames of HBV genes are shown in yellow. Black lines indicate spliced regions.

A

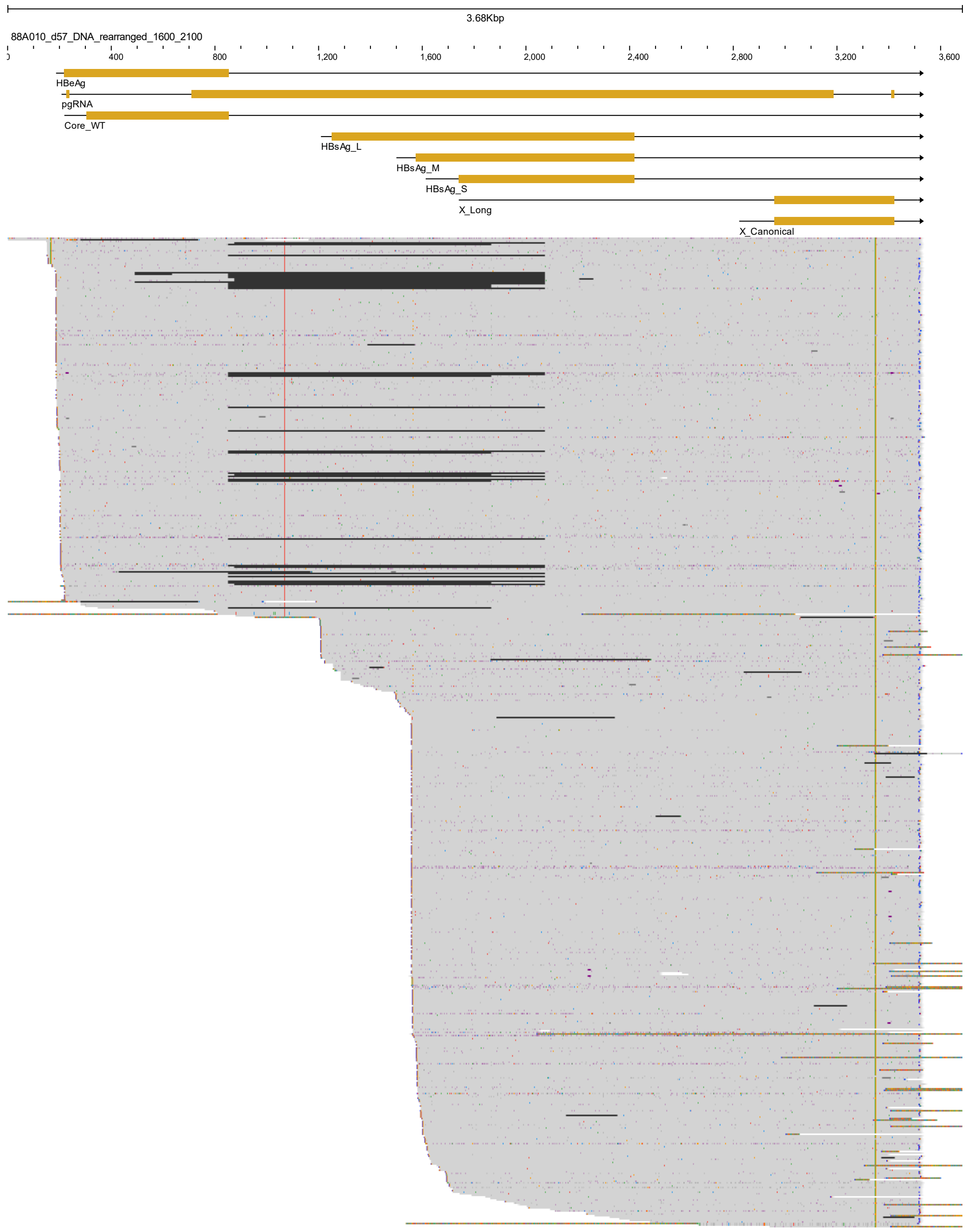

B

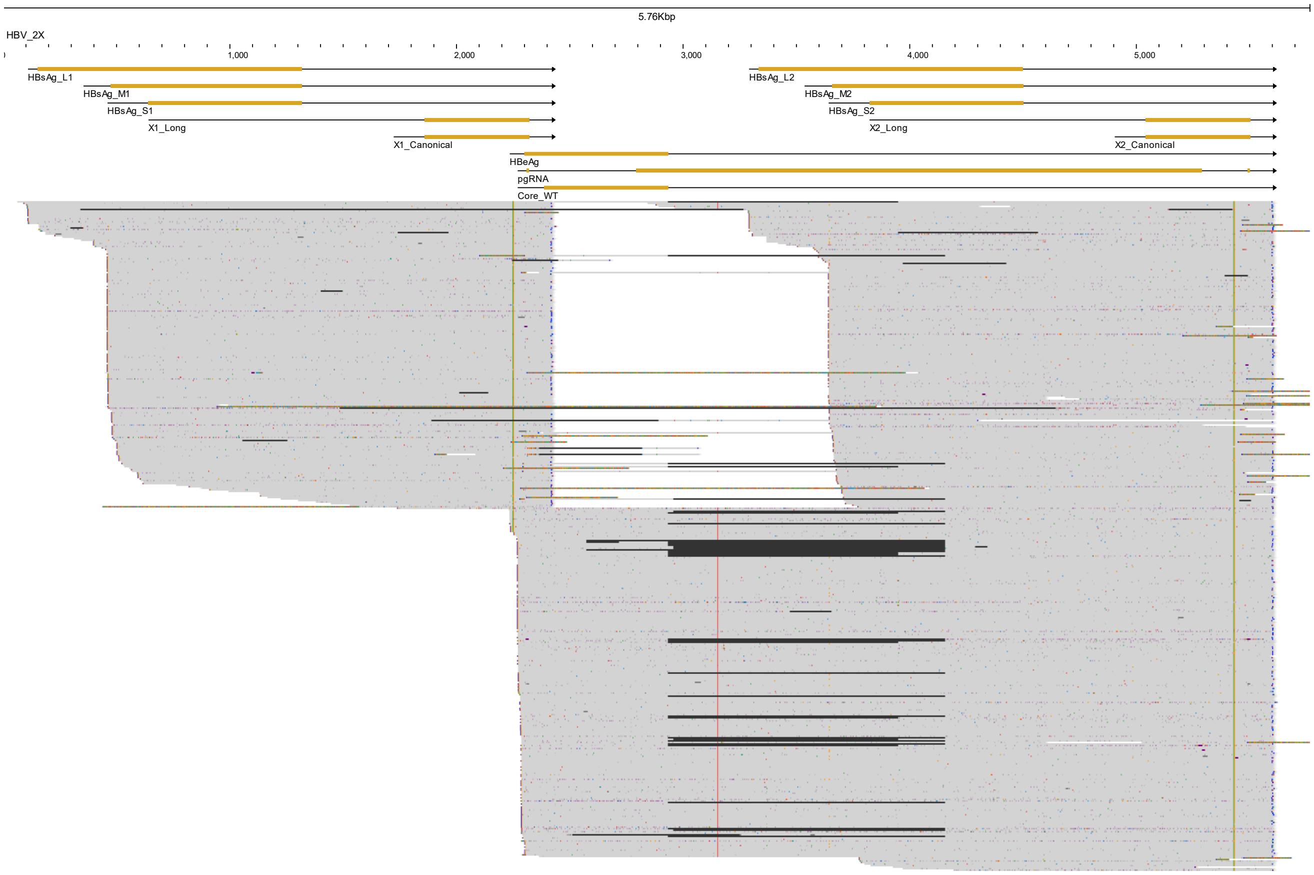

5.76Kbp
HBV_2X
1,000
2,000
3,000
4,000
5,000
HBsAg_L1
HBsAg_M1
HBsAg_S1
X1_Long
X1_Canonical
HBsAg_L2
HBsAg_M2
HBsAg_S2
X2_Long
X2_Canonical
HBeAg
pgRNA
Core_WT

Supplementary Figure S2. Chimpanzee HBV transcripts aligned by read start and read end positions on 2700–2100 concatemer and normalized transcript counts. Identified HBV transcripts aligned to the 2700–2100 concatemer are from HBeAg+ and HBeAg− chimpanzees: HBeAg+ chimpanzee A2A004 at study day 85 after the ETV lead-in (A, B), study day 323 that was two weeks after the final ARC-520 injection and one week after the final ETV dose (C, D), and study day 351 that was 33 days off all treatment (E, F); HBeAg+ chimpanzee A3A006 at HC (G, H), after ETV lead-in on day 141 (I, J) and 33 days off all treatment on study day 351 (K, L); and HBeAg+ chimpanzee A4A014 at HC (M, N). Pink bars, HBV PAS.

A

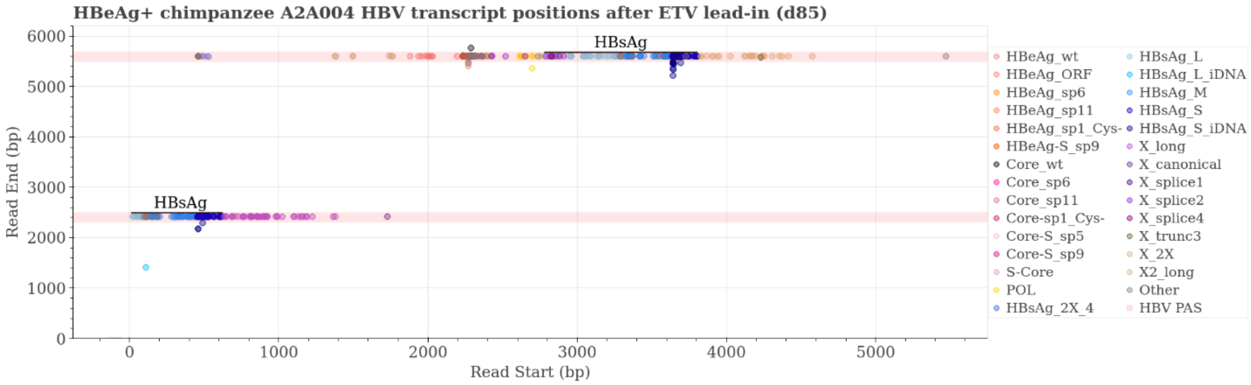

B

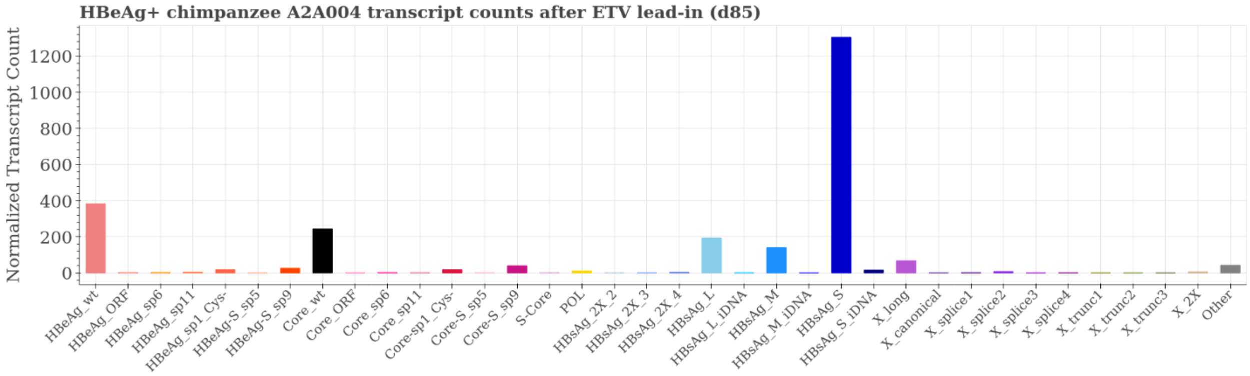

C

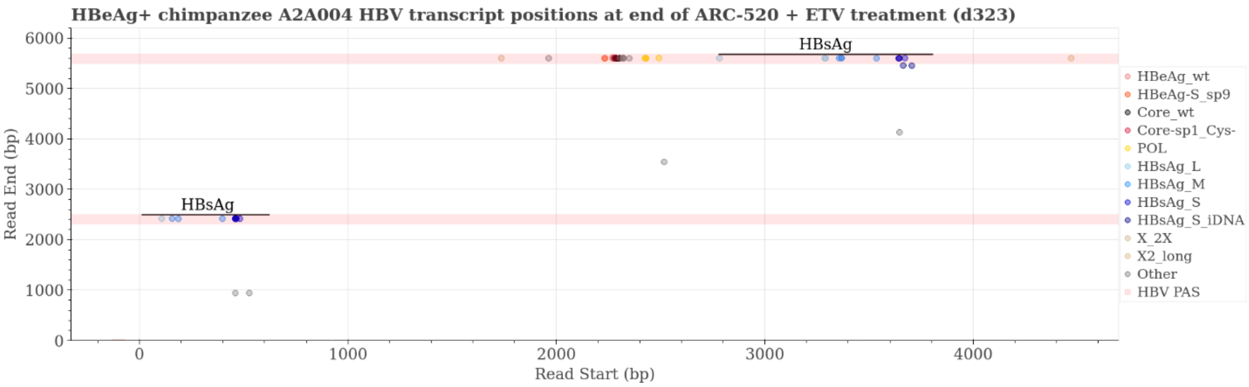
HBeAg+ chimpanzee A2A004 HBV transcript positions at end of ARC-520 + ETV treatment (d323)
HBsAg
HBsAg
Read End (bp)
Read Start (bp)
0
1000
2000
3000
4000
5000
6000
HBeAg_wt
HBeAg-S_sp9
Core_wt
Core-sp1_Cys-
POL
HBsAg_L
HBsAg_M
HBsAg_S
HBsAg_S_iDNA
X_2X
X2_long
Other
HBV PAS

D

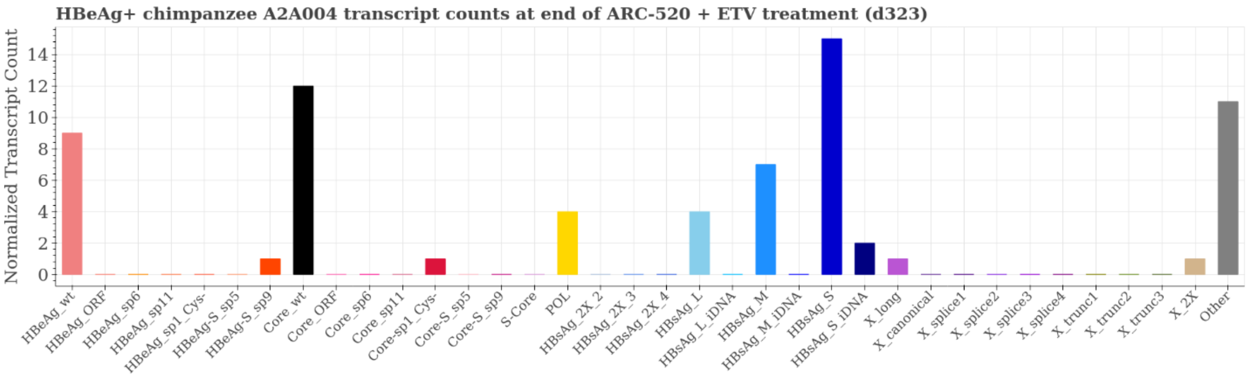
HBeAg+ chimpanzee A2A004 transcript counts at end of ARC-520 + ETV treatment (d323)
Normalized Transcript Count
0
2
4
6
8
10
12
14
HBeAg_wt
HBeAg_ORF
HBeAg_sp6
HBeAg_sp11
HBeAg_sp1_Cys-
HBeAg-S_sp5
HBeAg-S_sp9
Core_wt
Core_ORF
Core_sp6
Core_sp11
Core-sp1_Cys-
Core-S_sp5
Core-S_sp9
S-Core
POL
HBsAg_2X_2
HBsAg_2X_3
HBsAg_2X_4
HBsAg_L
HBsAg_L_iDNA
HBsAg_M
HBsAg_M_iDNA
HBsAg_S
HBsAg_S_iDNA
X_long
X_canonical
X_splice1
X_splice2
X_splice3
X_splice4
X_trunc1
X_trunc2
X_trunc3
X_2X
Other

E

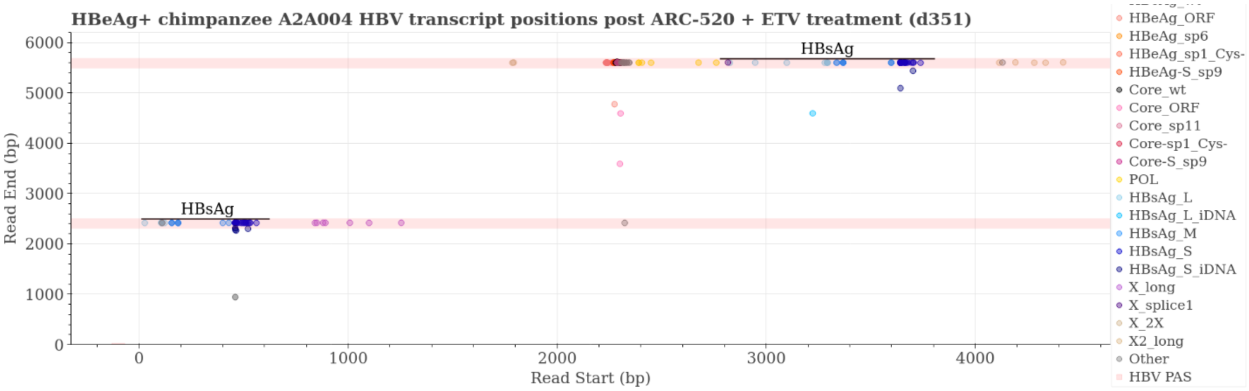
HBeAg+ chimpanzee A2A004 HBV transcript positions post ARC-520 + ETV treatment (d351)
HBsAg
HBsAg
Read End (bp)
Read Start (bp)
0
1000
2000
3000
4000
5000
6000
HBeAg_ORF
HBeAg_sp6
HBeAg_sp1_Cys-
HBeAg-S_sp9
Core_wt
Core_ORF
Core_sp11
Core-sp1_Cys-
Core-S_sp9
POL
HBsAg_L
HBsAg_L_iDNA
HBsAg_M
HBsAg_S
HBsAg_S_iDNA
X_long
X_splice1
X_2X
X2_long
Other
HBV PAS

F

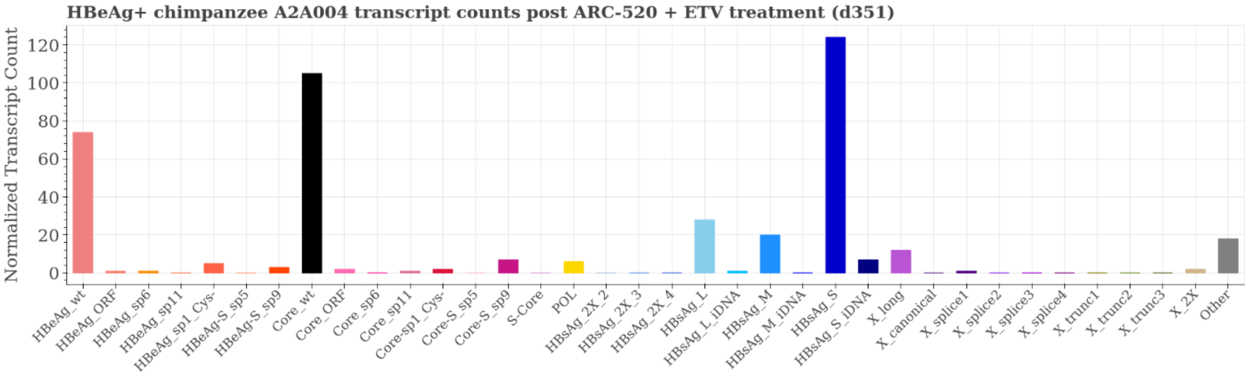

HBeAg+ chimpanzee A2A004 transcript counts post ARC-520 + ETV treatment (d351)
Normalized Transcript Count
0
20
40
60
80
100
120
HBeAg_wt
HBeAg_ORF
HBeAg_sp6
HBeAg_sp11
HBeAg_sp1_Cys-
HBeAg-S_sp5
HBeAg-S_sp9
Core_wt
Core_ORF
Core_sp6
Core_sp11
Core-sp1_Cys-
Core-S_sp5
Core-S_sp9
S-Core
POL
HBsAg_2X_2
HBsAg_2X_3
HBsAg_2X_4
HBsAg_L
HBsAg_L_iDNA
HBsAg_M
HBsAg_M_iDNA
HBsAg_S
HBsAg_S_iDNA
X_long
X_canonical
X_splice1
X_splice2
X_splice3
X_splice4
X_trunc1
X_trunc2
X_trunc3
X_2X
Other

G

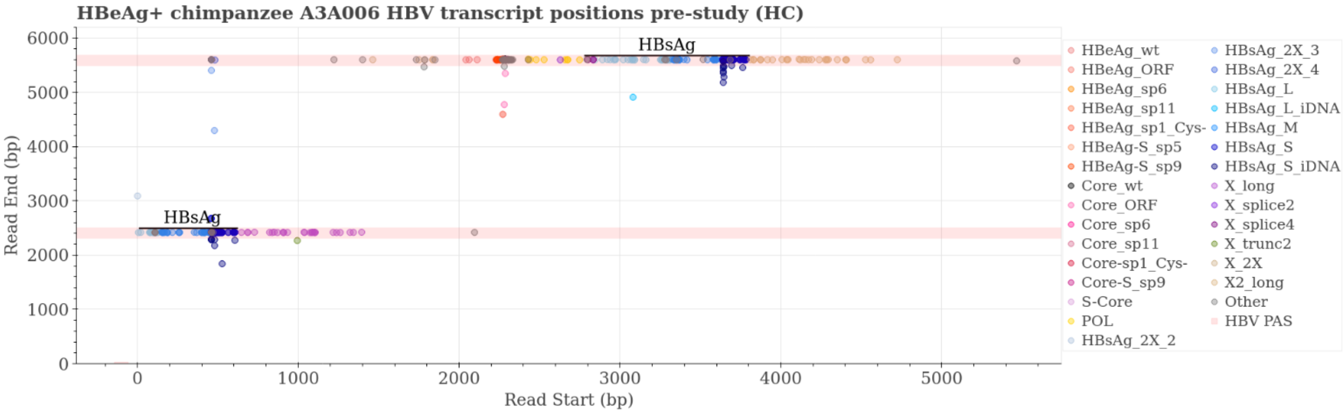

HBeAg+ chimpanzee A3A006 HBV transcript positions pre-study (HC)
HBsAg
HBsAg
Read End (bp)
0
1000
2000
3000
4000
5000
6000
Read Start (bp)
0
1000
2000
3000
4000
5000
HBeAg_wt
HBeAg_ORF
HBeAg_sp6
HBeAg_sp11
HBeAg_sp1_Cys-
HBeAg-S_sp5
HBeAg-S_sp9
Core_wt
Core_ORF
Core_sp6
Core_sp11
Core-sp1_Cys-
Core-S_sp9
S-Core
POL
HBsAg_2X_2
HBsAg_2X_3
HBsAg_2X_4
HBsAg_L
HBsAg_L_iDNA
HBsAg_M
HBsAg_S
HBsAg_S_iDNA
X_long
X_splice2
X_splice4
X_trunc2
X_2X
X2_long
Other
HBV PAS

H

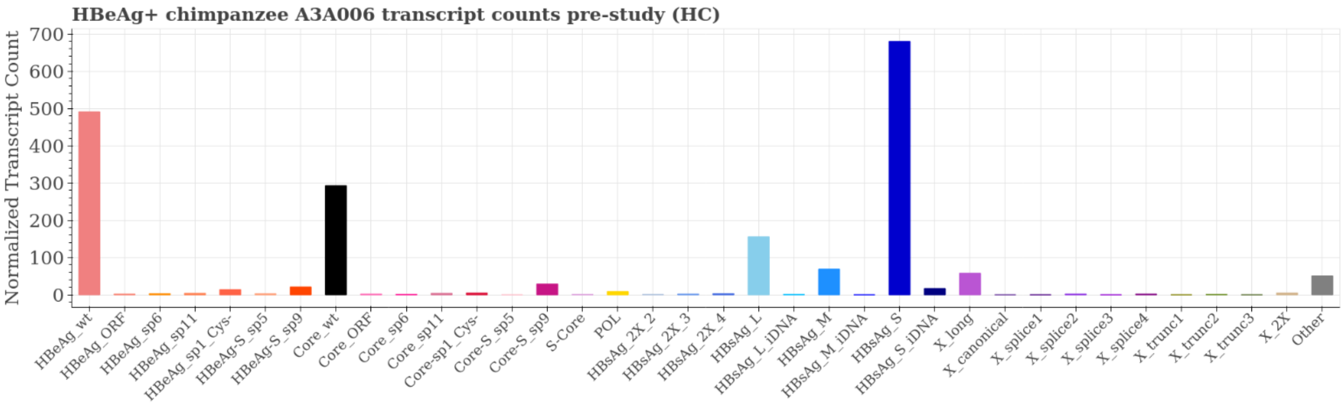

HBeAg+ chimpanzee A3A006 transcript counts pre-study (HC)
Normalized Transcript Count
0
100
200
300
400
500
600
700
HBeAg_wt
HBeAg_ORF
HBeAg_sp6
HBeAg_sp11
HBeAg_sp1_Cys-
HBeAg-S_sp5
HBeAg-S_sp9
Core_wt
Core_ORF
Core_sp6
Core_sp11
Core-sp1_Cys-
Core-S_sp5
Core-S_sp9
S-Core
POL
HBsAg_2X_2
HBsAg_2X_3
HBsAg_2X_4
HBsAg_L
HBsAg_L_iDNA
HBsAg_M
HBsAg_M_iDNA
HBsAg_S
HBsAg_S_iDNA
X_long
X_canonical
X_splice1
X_splice2
X_splice3
X_splice4
X_trunc1
X_trunc2
X_trunc3
X_2X
Other

I

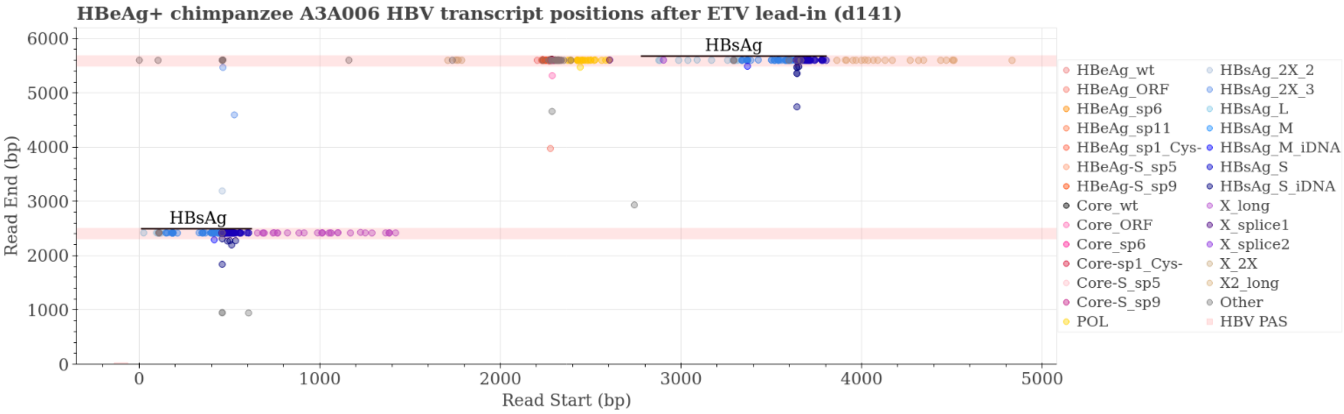
HBeAg+ chimpanzee A3A006 HBV transcript positions after ETV lead-in (d141)
HBsAg
HBsAg
Read End (bp)
Read Start (bp)
HBeAg_wt
HBeAg_ORF
HBeAg_sp6
HBeAg_sp11
HBeAg_sp1_Cys-
HBeAg-S_sp5
HBeAg-S_sp9
Core_wt
Core_ORF
Core_sp6
Core-sp1_Cys-
Core-S_sp5
Core-S_sp9
POL
HBsAg_2X_2
HBsAg_2X_3
HBsAg_L
HBsAg_M
HBsAg_M_iDNA
HBsAg_S
HBsAg_S_iDNA
X_long
X_splice1
X_splice2
X_2X
X2_long
Other
HBV PAS

J

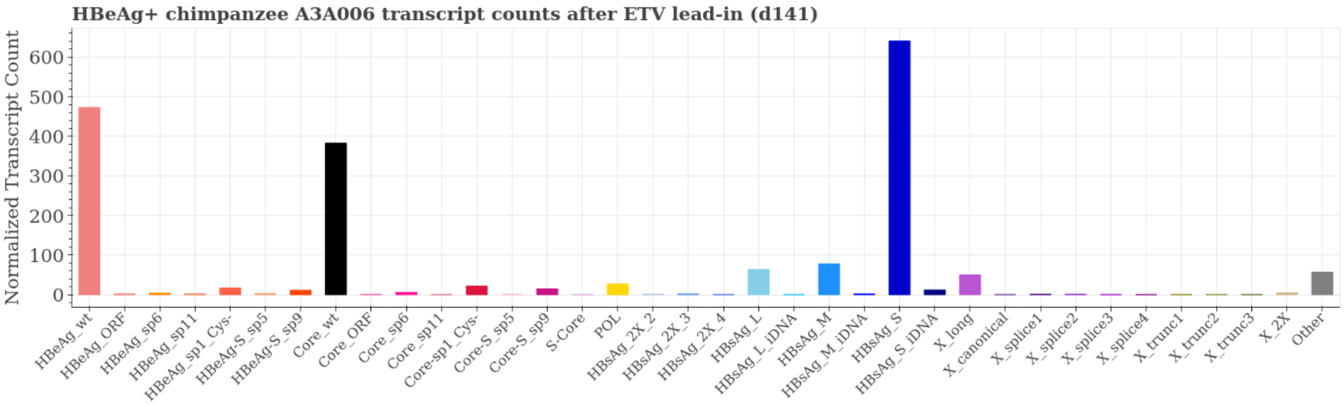
HBeAg+ chimpanzee A3A006 transcript counts after ETV lead-in (d141)
Normalized Transcript Count
HBeAg_wt
HBeAg_ORF
HBeAg_sp6
HBeAg_sp11
HBeAg_sp1_Cys-
HBeAg-S_sp5
HBeAg-S_sp9
Core_wt
Core_ORF
Core_sp6
Core_sp11
Core-sp1_Cys-
Core-S_sp5
Core-S_sp9
S-Core
POL
HBsAg_2X_2
HBsAg_2X_3
HBsAg_2X_4
HBsAg_L
HBsAg_L_iDNA
HBsAg_M
HBsAg_M_iDNA
HBsAg_S
HBsAg_S_iDNA
X_long
X_canonical
X_splice1
X_splice2
X_splice3
X_splice4
X_trunc1
X_trunc2
X_trunc3
X_2X
Other

K

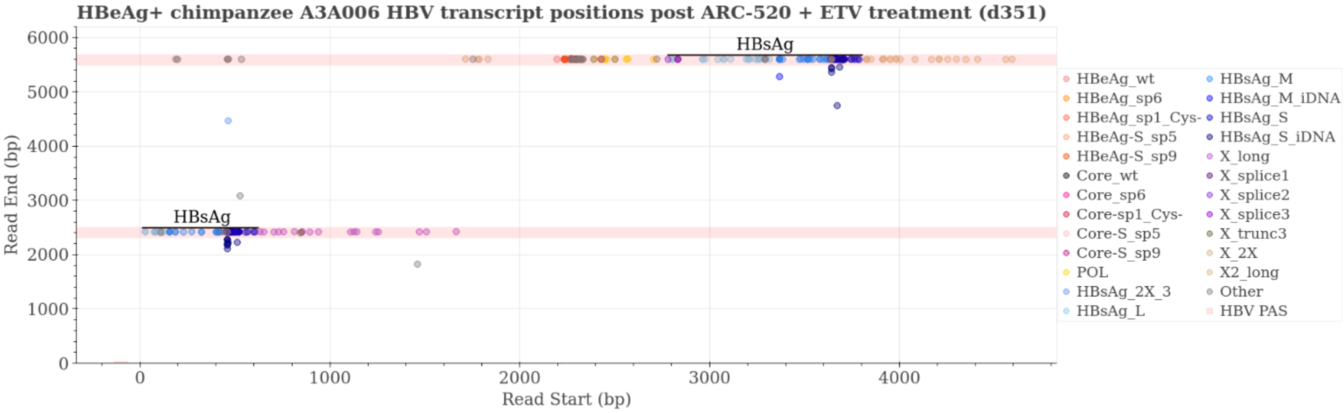
HBeAg+ chimpanzee A3A006 HBV transcript positions post ARC-520 + ETV treatment (d351)
HBsAg
HBsAg
Read End (bp)
Read Start (bp)
HBeAg_wt
HBeAg_sp6
HBeAg_sp1_Cys-
HBeAg-S_sp5
HBeAg-S_sp9
Core_wt
Core_sp6
Core-sp1_Cys-
Core-S_sp5
Core-S_sp9
POL
HBsAg_2X_3
HBsAg_L
HBsAg_M
HBsAg_M_iDNA
HBsAg_S
HBsAg_S_iDNA
X_long
X_splice1
X_splice2
X_splice3
X_trunc3
X_2X
X2_long
Other
HBV PAS

L

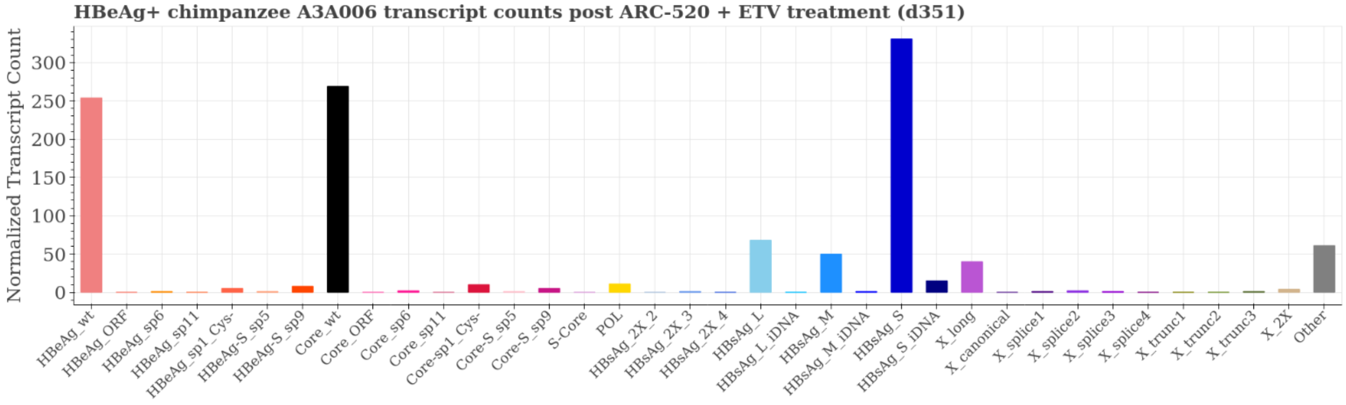
HBeAg+ chimpanzee A3A006 transcript counts post ARC-520 + ETV treatment (d351)
Normalized Transcript Count
HBeAg_wt
HBeAg_ORF
HBeAg_sp6
HBeAg_sp11
HBeAg_sp1_Cys-
HBeAg-S_sp5
HBeAg-S_sp9
Core_wt
Core_ORF
Core_sp6
Core_sp11
Core-sp1_Cys-
Core-S_sp5
Core-S_sp9
S-Core
POL
HBsAg_2X_2
HBsAg_2X_3
HBsAg_2X_4
HBsAg_L
HBsAg_L_iDNA
HBsAg_M
HBsAg_M_iDNA
HBsAg_S
HBsAg_S_iDNA
X_long
X_canonical
X_splice1
X_splice2
X_splice3
X_splice4
X_trunc1
X_trunc2
X_trunc3
X_2X
Other

M

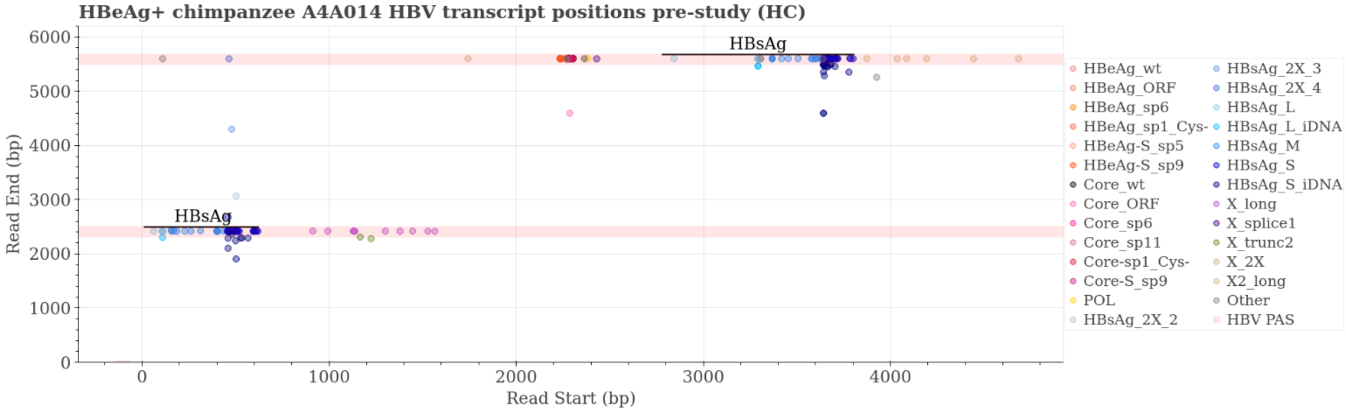
HBeAg+ chimpanzee A4A014 HBV transcript positions pre-study (HC)
HBsAg
HBsAg
Read End (bp)
Read Start (bp)
HBeAg_wt
HBeAg_ORF
HBeAg_sp6
HBeAg_sp1_Cys-
HBeAg-S_sp5
HBeAg-S_sp9
Core_wt
Core_ORF
Core_sp6
Core_sp11
Core-sp1_Cys-
Core-S_sp9
POL
HBsAg_2X_2
HBsAg_2X_3
HBsAg_2X_4
HBsAg_L
HBsAg_L_iDNA
HBsAg_M
HBsAg_S
HBsAg_S_iDNA
X_long
X_splice1
X_trunc2
X_2X
X2_long
Other
HBV PAS

N

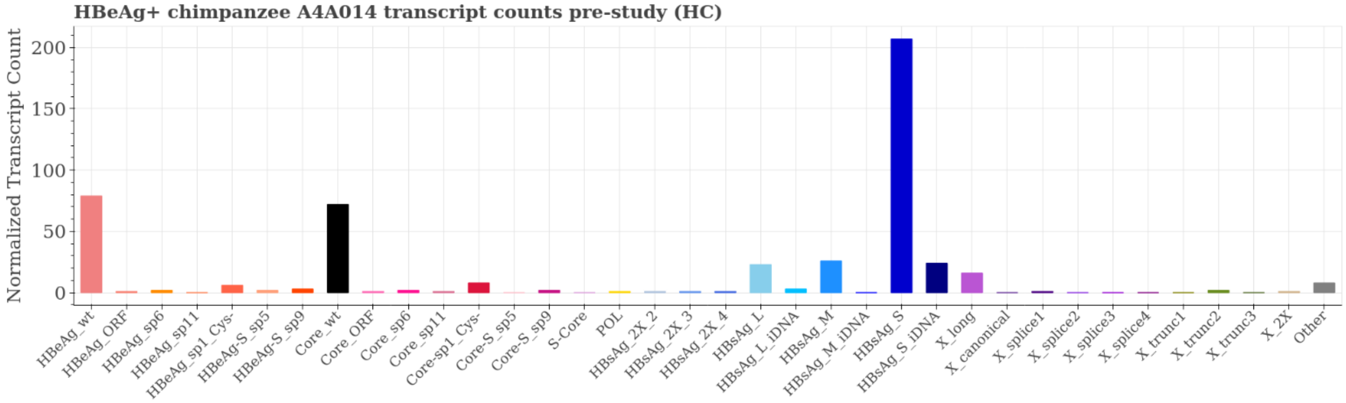
HBeAg+ chimpanzee A4A014 transcript counts pre-study (HC)
Normalized Transcript Count

Supplementary Figure S3. Sequences of first (A) and second (B) HBx open reading frames in the X_2X transcript of chimpanzee A3A006, study day 141 after the ETV lead-in. The upper line sequence is that of the chimpanzee reference genome 88A010. The sequence highlighted in green is the sequence of the transcript. The translated sequence is show in yellow.

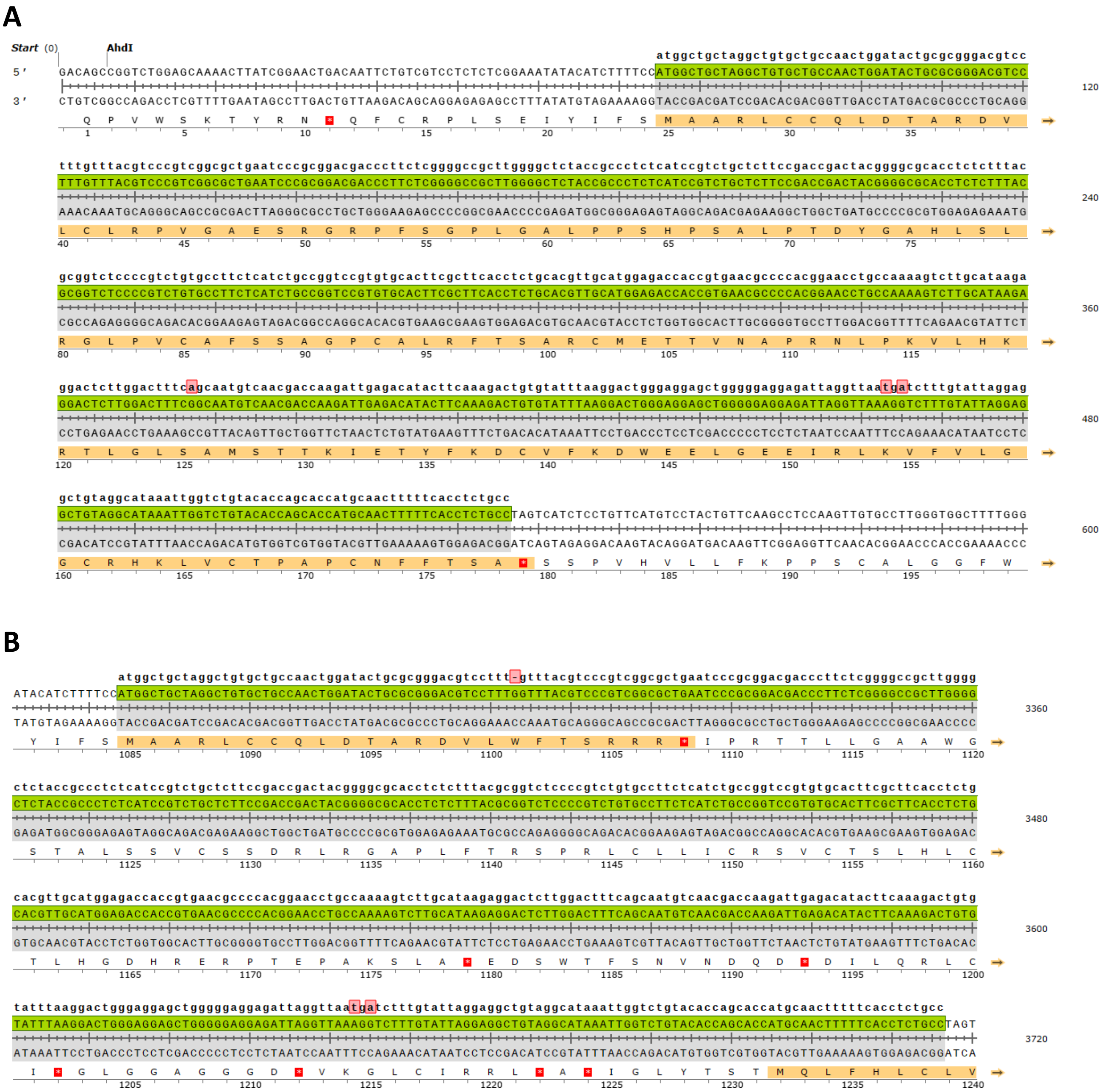

Supplementary Figure S4. Alignment of Iso-seq HBV transcripts from Heparc-2001 study patients. HBV transcripts from patients 705, 710 and 712 were aligned to the 2700–2200 concatemer of GenBank accession number AB073827. Above each alignment of transcripts is the coverage map. Blue mark indicates the most frequent transcript termination site. Arrow at end of transcripts indicates HBV PAS.

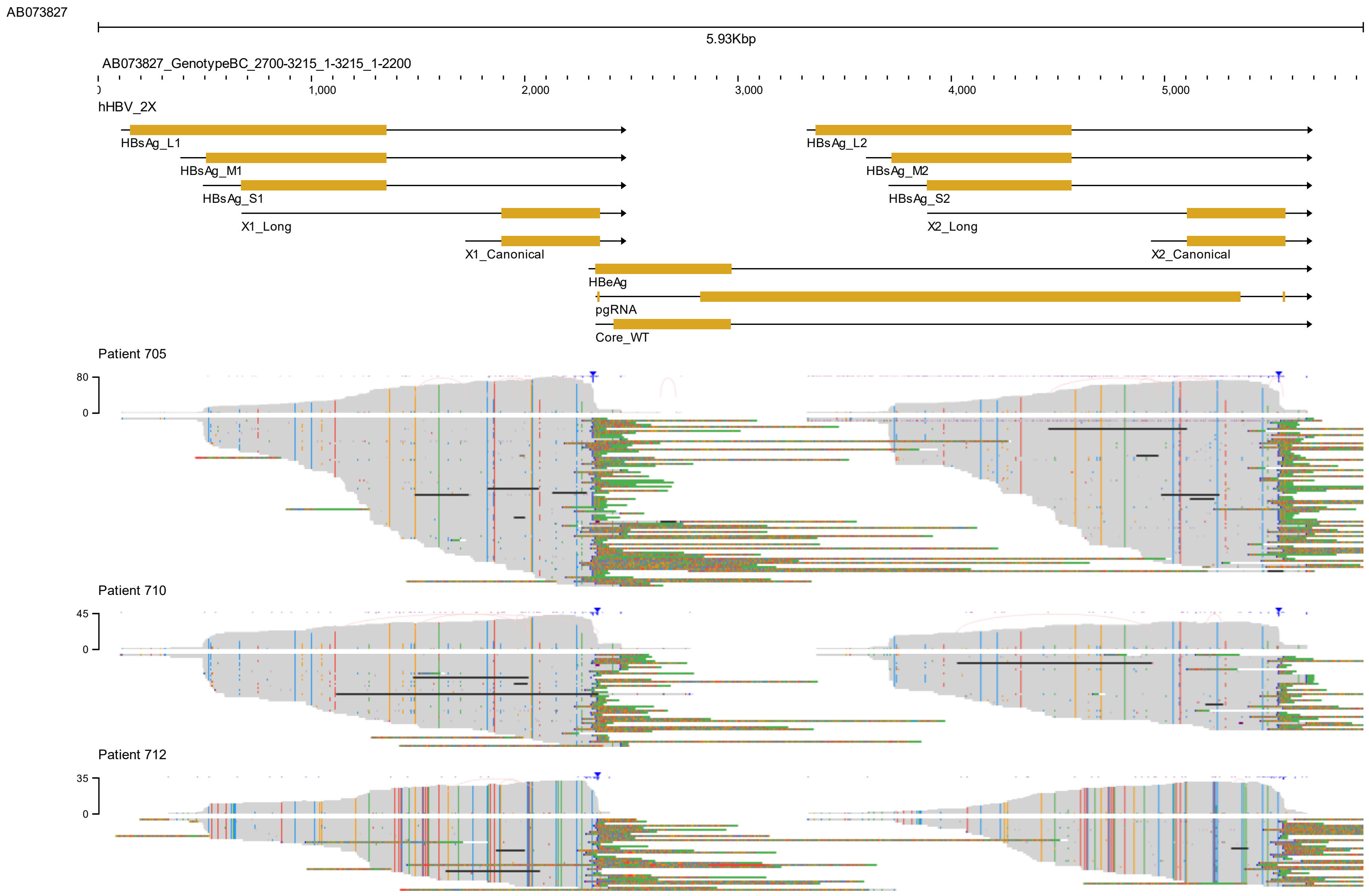
AB073827
5.93Kbp
AB073827_GenotypeBC_2700-3215_1-3215_1-2200
1,000
2,000
3,000
4,000
5,000
hHBV_2X
HBsAg_L1
HBsAg_M1
HBsAg_S1
X1_Long
X1_Canonical
HBsAg_L2
HBsAg_M2
HBsAg_S2
X2_Long
X2_Canonical
HBeAg
pgRNA
Core_WT
Patient 705
80
0
Patient 710
45
0
Patient 712
35
0

References

1. Wooddell, C.I., et al., *RNAi-based treatment of chronically infected patients and chimpanzees reveals that integrated hepatitis B virus DNA is a source of HBsAg.* Sci Transl Med, 2017. **9**(409).
2. Werle-Lapostolle, B., et al., *Persistence of cccDNA during the natural history of chronic hepatitis B and decline during adefovir dipivoxil therapy.* Gastroenterology, 2004. **126**(7): p. 1750-8.
3. Wong, D.K., et al., *Reduction of hepatitis B surface antigen and covalently closed circular DNA by nucleos(t)ide analogues of different potency.* Clin Gastroenterol Hepatol, 2013. **11**(8): p. 1004-10 e1.
4. Lai, C.L., et al., *Reduction of covalently closed circular DNA with long-term nucleos(t)ide analogue treatment in chronic hepatitis B.* J Hepatol, 2017. **66**(2): p. 275-281.
5. Rodriguez-Frias, F., et al., *Quasispecies structure, cornerstone of hepatitis B virus infection: mass sequencing approach.* World J Gastroenterol, 2013. **19**(41): p. 6995-7023.
6. Yuen, M.F., et al., *Long-term serological, virological and histological responses to RNA inhibition by ARC-520 in Chinese chronic hepatitis B patients on entecavir treatment.* Gut, 2022. **71**(4): p. 789-797.
